# Linear infrastructure habitats increase landscape-scale diversity of plants but not of flower-visiting insects

**DOI:** 10.1038/s41598-020-78090-y

**Published:** 2020-12-07

**Authors:** Juliana Dániel-Ferreira, Riccardo Bommarco, Jörgen Wissman, Erik Öckinger

**Affiliations:** 1 Department of Ecology, Swedish University of Agricultural Sciences, Box 7044, 75007 Uppsala, Sweden; 2 Swedish Biodiversity Centre, CBM, Swedish University of Agricultural Sciences, Box 7016, 75007 Uppsala, Sweden

**Keywords:** Ecology, Biodiversity, Community ecology, Conservation biology

## Abstract

Habitats along linear infrastructure, such as roads and electrical transmission lines, can have high local biodiversity. To determine whether these habitats also contribute to landscape-scale biodiversity, we estimated species richness, evenness and phylogenetic diversity of plant, butterfly and bumblebee communities in 32 4 km^2^ landscapes with or without power line corridors, and with contrasting areas of road verges. Landscapes with power line corridors had on average six more plant species than landscapes without power lines, but there was no such effect for butterflies and bumblebees. Plant communities displayed considerable evenness in species abundances both in landscapes with and without power lines and high and low road verge densities. We hypothesize that the higher number of plant species in landscapes with power line corridors is due to these landscapes having a higher extinction debt than the landscapes without power line corridors, such that plant diversity is declining slower in landscapes with power lines. This calls for targeted conservation actions in semi-natural grasslands within landscapes with power line corridors to maintain biodiversity and prevent imminent population extinctions.

## Introduction

The area of semi-natural grasslands, i.e. grasslands that are traditionally managed by mowing or grazing, without any input of fertilizers or herbicides^1^, has been steadily declining due mostly to afforestation and silviculture occurring during the twentieth century^2^. In Sweden, traditionally managed semi-natural grasslands are one of the most species-rich habitats but the decrease in area during the last 80 to 90 years has been estimated to be around 90%^1,3^. Currently, the area of semi-natural grasslands of high nature value in the country is estimated to be around 2986.9 km^2^ according to the Swedish National survey of semi-natural pastures and meadows (TUVA: http://www.jordbruksverket.se/tuva; access date: 06-08-2020). This has led to drastic declines of species coupled to these habitats^4–6^. Additionally, a reduction of habitat area entails altered dominance patterns in communities, such that already common species become even more dominant and rare species decrease in abundance^6,7^. This can in turn contribute to local and regional extinctions by altering species interactions which may disrupt ecosystem functioning^7–9^.

Given the appropriate abiotic conditions, the plant species composition in verges along linear transportation infrastructure such as railways, roads and power line corridors, can at least to some extent resemble the vegetation in traditionally managed semi-natural grasslands^10–12^. These linear infrastructure habitats are thus novel habitats (i.e. anthropogenically managed habitats with natural analogues^13^) managed to be kept open and in an early successional stage, which can provide important resources for many plants and insects. Linear landscape features have been shown to facilitate both the spread of native and non-native species throughout the landscape^14–17^. Furthermore, linear infrastructure itself, such as roads, have well documented negative effects on biodiversity such as traffic mortality and pollution^18–20^. Yet, rare and threatened species of plants and invertebrates have been documented in these novel habitats^12,21–24^. Therefore, we need to establish the net effects of linear infrastructure habitats on biodiversity.

Linear infrastructure habitats cover enormous areas globally, often surpassing the area of remnant natural habitats^21,25,26^. They have also been shown to be able to harbour just as much biodiversity locally as their counterparts^21,24^. However, the question whether this high local diversity also translates to higher landscape-level biodiversity in landscapes with large areas of linear infrastructure habitats remains unanswered^21^. More generally, species richness within a certain habitat typically increases with area, but it remains unclear if the effect of area on diversity at the patch scale (α diversity) is also observed at the landscape scale (ϒ diversity, i.e., data pooled among sites within a landscape^27^)^28^. As long linear habitats, linear infrastructure habitats also have the potential to increase ϒ diversity through increased landscape connectivity. Increasing area and connectivity in the landscape should then result in an increase in α and ϒ diversity, especially for grassland species as they are greatly influenced by the regional species pool because they are subject to frequent disturbances^29,30^. If contemporary linear infrastructure habitats indeed contribute to landscape level biodiversity in addition to diverse semi-natural grassland habitats, then they constitute a potentially great but currently poorly utilized opportunity for biodiversity conservation^21^. Managing linear infrastructure habitats for promoting diversity of plants and pollinators, and not only for infrastructure maintenance, can be crucial if their core habitat continues to diminish^11,31^.

Traditionally, conservation research and management has focused strongly on species richness but recently other aspects of biodiversity have received increased attention. One reason is that there is growing evidence that ecosystem functioning is often not best predicted by the number of species in a community, but is instead more related to other dimensions of biodiversity such as phylogenetic diversity, functional diversity or community evenness (e.g.^32^). We argue that it is important to determine the effects of linear infrastructure habitats at the landscape scale on the three most essential aspects of diversity: richness, evenness and evolutionary distinctiveness^33,34^. Evolutionary distinctiveness takes into account how similar or different the species in a community are based on their evolutionary relationships (i.e. the distance between species in the evolutionary tree), and it can be quantified by estimating the phylogenetic diversity of the community^34^. Provided that an increase in habitat area and connectivity in the landscape leads to an increase in species richness and abundance^35,36^, then the probability of rare species occurring in the community would also increase. If these rare species are not closely related to species that already occur in the community, then this would result in a higher phylogenetic diversity than that expected by chance. This information could potentially be useful to predict and prevent further extinctions if evolutionarily similar species are being systematically threatened by anthropogenically driven changes in the landscape.

Our aim was to investigate the relationship between the amount of linear infrastructure habitats and of semi-natural grasslands in 2 × 2 km landscapes with the species richness, evenness, and phylogenetic diversity of plants, butterflies and bumblebees. Assuming that an increase in the area of linear infrastructure habitats translates into both an increase in grassland habitat area in the landscape and in an increase in landscape connectivity for both plants and flower-visiting insects, we predicted that: (1) a high proportion of linear infrastructure habitats in the landscape will have a positive effect on the number of species of plants, bumblebees and butterflies in the landscape, (2) an increase in the area of linear infrastructure habitats, resulting in higher landscape connectivity, will increase the community evenness of plants, bumblebees and butterflies in the landscape, and (3) an increase in area of linear infrastructure habitats will have a positive effect on the phylogenetic diversity of plants, bumblebees and butterflies in the landscape. In other words, we expect a positive relationship between all three aspects of diversity and the area of linear infrastructure habitats in the landscape.

## Results

A total of 2 704 butterfly individuals belonging to 51 species, 1 316 bumblebee individuals belonging to 19 species and 128 species of plants were recorded within the three habitats (semi-natural grasslands and road verges of small and big roads) in the 32 landscapes. The most abundant butterfly species was *Aphantopus hyperantus* (974 individuals), followed by *Coenonympha arcania* (281 individuals), *Thymelicus lineola* (178 individuals) and *Pieris napi* (167 individuals). The most abundant bumblebee species was *Bombus pascuorum* (347 individuals), *B. lucorum* (170 individuals) and *B. terrestris* (157 individuals). The most common plant species were *Achillea millefolium*, *Agrostis capillaris*, *Dactylis glomerata* and *Taraxacum vulgare* (Supplementary Figures S4–S6).

### Species richness

**Figure 1:**
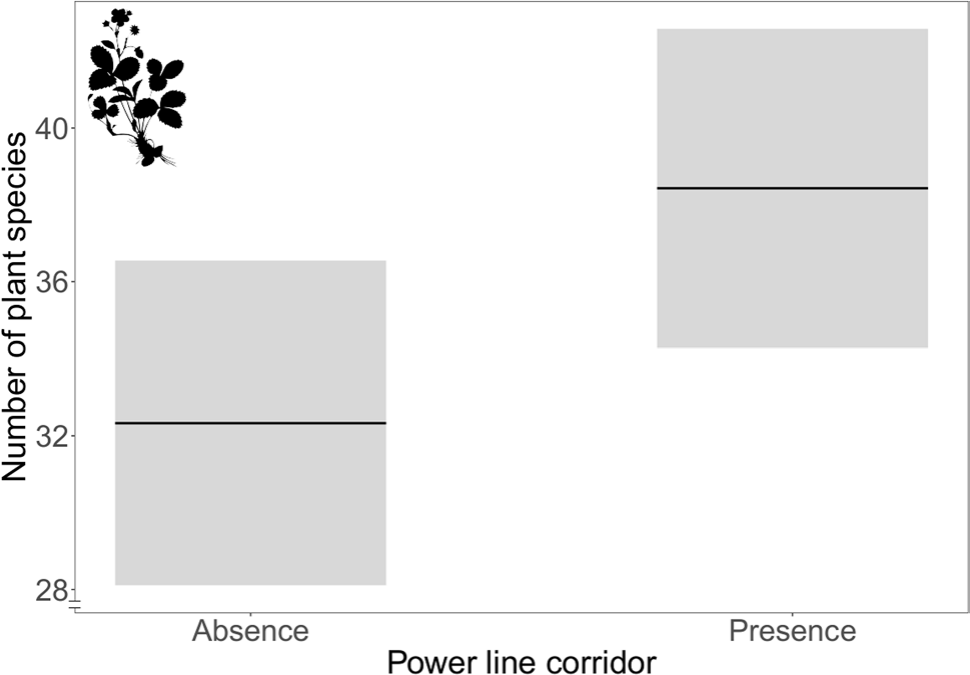
Species richness of plant species is in average ~ 6 species higher in landscapes with power line corridors than in landscapes without power line corridors. The grey bands represent the 95% confidence intervals. Note that the y-axis does not start at zero. The silhouette image was available under Public Domain license at PhyloPic (http://phylopic.org).

Landscapes with power line corridors had on average ~ 6 (± 2.36) more plant species than landscapes without power line corridors (Fig. 1; t-value = 2.6, *p* = 0.01). There was no relationship between the amount of linear infrastructure habitats and the number of butterfly or bumblebee species in the landscape, or between the amount of linear infrastructure and the number of indicator plant species. For the two insect groups, the models with the lowest AICc did not include any interaction between the explanatory variables. However, for butterflies the model with an interaction between the power line corridors and the road verge density performed almost as well as the model without any interactions (ΔAICc = 0.90). For plants and indicator plant species, the best model (with the lowest AICc) was the model with main effects but without interacting explanatory variables (Supplementary Table S6). There was no relationship between the amount of semi-natural grasslands in the landscape and species richness of any of the studied organisms (Supplementary Table S7).

### Species composition

**Figure 2:**
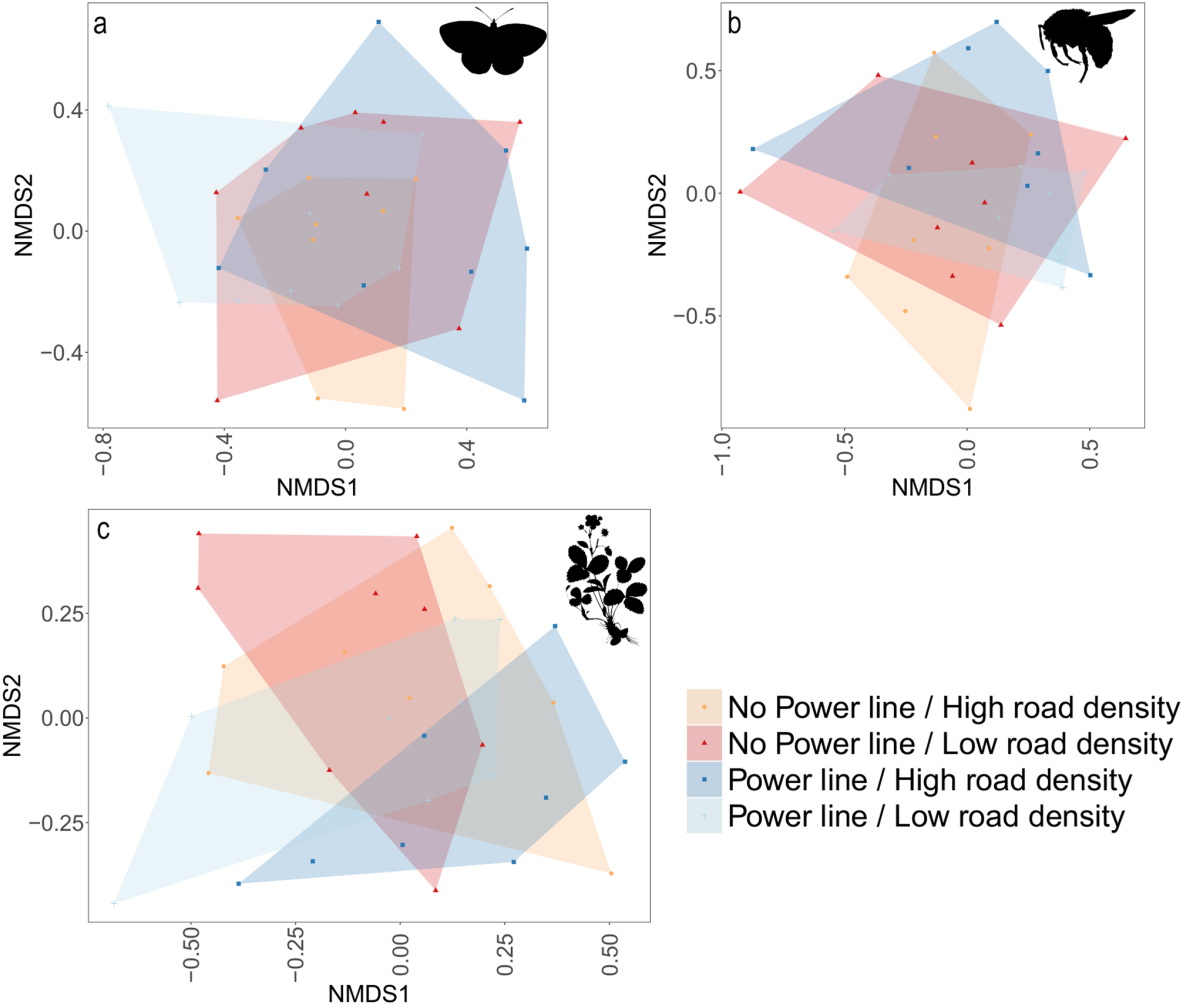
Non-metric multidimensional scaling (NMDS) analysis for butterflies (**a**; stress = 0.23), bumblebees (**b**; stress = 0.24) and plants (**c**; stress = 0.24) in the four landscape categories. There are not evident differences in the species compositions between landscape categories. See Supplementary Fig. S1–S3 for figures including species positions. Silhouette images were available under Public Domain license at PhyloPic (http://phylopic.org).

The ordination analysis did not show any clear clustering of species in any of the landscape categories, indicating that most species are present in all of the landscape categories (Fig. 2).

### Evenness

**Figure 3:**
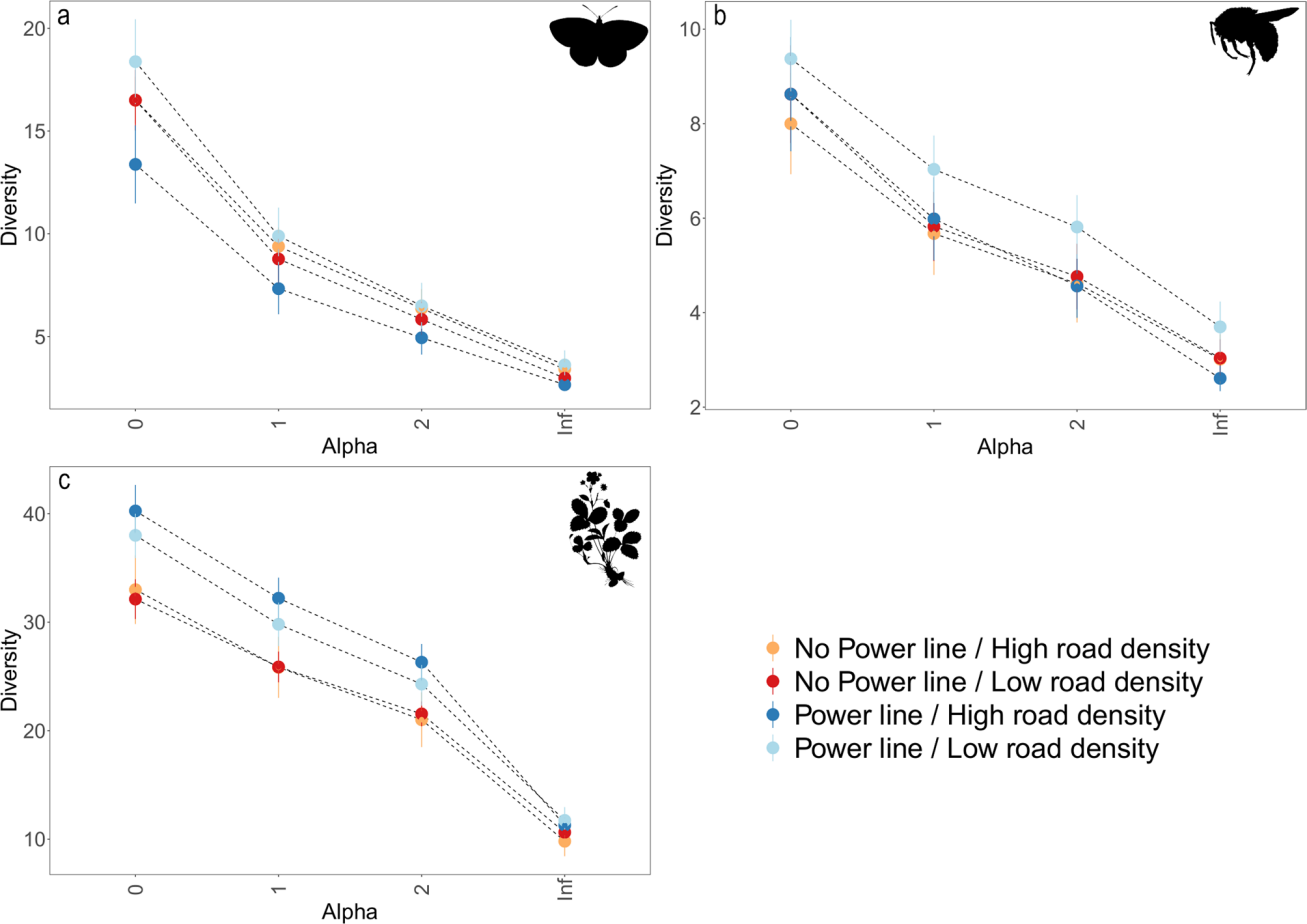
Diversity profiles for butterflies (**a**), bumblebees (**b**), and plants (**c**). Each value of alpha represents a different biodiversity index: 0 = species richness, 1 = Shannon diversity, 2 = Simpson diversity and Inf = Berger-Parker. The indexes put different weights to rare and common species (i.e. rare species have the same weight as common species in alpha = 0 while Berger-Parker ignores rare species). Diversity is represented in Hill numbers, meaning that all indexes predict number of species and can thus be compared to each other. The bars represent the standard error of the mean. Note that the connections between points do not represent a relationship between indexes. Silhouette images were available under Public Domain license at PhyloPic (http://phylopic.org).

In general, the diversity profiles show that all landscape categories had similar mean abundance distributions for all three organism groups. Butterfly communities in the four landscape categories had on average low evenness and bumblebee communities had also on average low evenness in all landscape categories, but to a lesser extent than for the butterflies (Fig. 3a, b). Plant communities in the four landscape categories displayed higher evenness compared to the flower-visiting insect communities (Fig. 3c).

**Figure 4:**
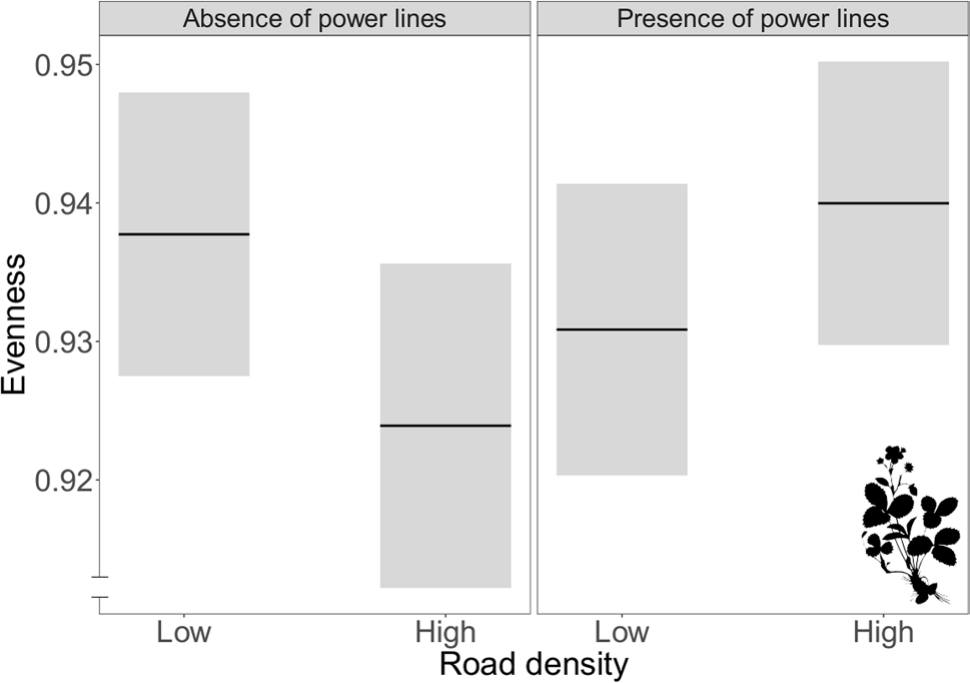
Interaction effect on the evenness (E_Shannon_) of plant species in the landscape. In landscapes with no power line corridors, an increase in road verge density has a negative effect on the abundance distribution of plant species. In contrast, in landscapes with power line corridors, an increase in road verge density has a positive effect on the abundance distribution of plant species. Grey bars represent the 95% confidence intervals. The silhouette image was available under Public Domain license at PhyloPic (http://phylopic.org).

In landscapes without power line corridors, an increasing proportion of road verges gave lower evenness of plant species. On the other hand, the evenness of plant species increased with an increasing proportion of road verges in the landscape when power line corridors were present (Fig. 4; estimate ± SE: 0.023 ± 0.01, t-value = 2.1, *p* = 0.04*). Overall, plant communities at the landscape level had an even abundance distribution according E_Shannon_, where the mean evenness in all 32 landscapes was 0.93 (± 0.01), with a minimum value of 0.90 and a maximum of 0.96.

There was no association between the amount of grasslands in the landscape and the evenness of any of the studied organisms (Table S6–S7).

### Phylogenetic diversity

There was no relation between the amount of linear infrastructure habitats or the amount of grasslands in the landscape and the standardized phylogenetic diversity of butterflies, plants or bumblebees (Table S6–S7). For all of the organism groups, the best model was the simplest; where no interactions between explanatory variables were included. However, for butterflies and plants there was no difference (ΔAICc < 2) between the simplest model and the model including an interaction between the power line corridors and the road verge density in the landscape. For bumblebees, the simplest model was clearly the best one. There was no correlation between the standardized effect size of phylogenetic diversity and species richness of any of the organism groups (Pearson: butterflies r = -0.23, *p* = 0.2; bumblebees r = 0.24, *p* = 0.19; plants r = − 0.09, *p* = 0.63).

## Discussion

Landscapes containing power line corridors had on average six more plant species, and had higher evenness of plants than landscapes without power line corridors. Yet, we found no such relationships for the diversity of butterflies or bumblebees, and road verges did not affect the diversity of any of the studied taxa. These results are unexpected, given that we expected a positive relationship between both types of linear infrastructure habitats for all organism groups and all aspects of diversity and previous findings show that these habitats can have a high local species diversity^21,24^. The positive relationship between power line corridors and the evenness and number of plant species in the landscape did, however, not translate to an increase in their phylogenetic diversity. Linear infrastructure habitats appear not to have a strong overall effect on grassland biodiversity at the landscape scale, despite their high local diversity^11,22,23^.

Neither power line corridors nor road verge density affected species composition of any of the taxa as shown by the ordination analysis. Hence, the positive relationship between power line corridors and the number of plant species on a landscape scale cannot be explained by this habitat adding another set of species to the landscape. Instead, we see two alternatives, but not mutually exclusive, explanations for this pattern. First, the presence of power line corridors implies a larger total grassland area in the landscape, which could explain why such landscapes supported a higher number of plant species^36^. However, if this was the main explanation, we would have expected to find similar patterns for butterflies, bumblebees and for indicator plant species^35,36^. Indicator plant species as a functional group was intended to give a more clear response as it does not include the noise from other species that have the ability to establish in other habitats in the landscape. Although previous studies have found high numbers of flower-visiting insect species in power line corridors^12,37^, we found no increase in landscape-scale diversity of butterflies or bumblebees in landscapes with power line corridors.

Secondly, if landscapes are losing biodiversity due to a loss of grassland area in the past^2,38^, the additional habitat area provided by the power line corridors might be slowing down the loss of plant species from these landscapes. The time lag between habitat loss and biodiversity response increases with increasing habitat area and decreasing isolation, which in this case can be provided by power line corridors^38^. Therefore, we argue that landscapes with power line corridors might have a higher extinction debt than landscapes without. Since short-lived species such as insects are expected to respond faster to habitat loss than long-lived organisms such as most grassland plants^39^, this could also explain the contrasting patterns of plant and insect species richness, i.e., the insects have already paid the debt. An emerging issue with these two arguments could be that if an increase in grassland habitat area increases landscape-scale diversity or extends the relaxation time of vascular plants then a similar effect of the area of road verges in the landscape is expected. However, we found no relationship between the area of road verges and the species richness of plants in the landscape.

Nonetheless, there are a number of differences between road verges and power line corridors that could explain this discrepancy. One of the most fundamental differences between these linear infrastructure habitats is management, resulting in partly different plant communities (unpublished data). In southern Sweden, road verges are mowed between one and three times a year whereas power line corridors are managed in an eight-year cycle^40^. The frequency and timing of management has been previously shown to have an effect on the species richness, composition, seed rain and establishment of plants in the road verges and adjacent habitats^41,42^. Furthermore, the vegetation in road verges is subjected to additional sources of disturbance such as pollution from road maintenance and passing vehicles^43,44^. Roads can also increase the rate of mortality and predation, or act as strong barriers to movement and reduce landscape connectivity^20,24,45^. In this context, it is important to highlight that even though we found no positive effect of the amount of road verges on landscape-scale biodiversity, there was no negative effect of a high road density either. Hence, it is possible that any negative effects from the roads and traffic on landscape-scale biodiversity were counterbalanced by the increased area of grassland habitat in the road verges.

We found no relationship between the area of semi-natural grasslands in the landscape and diversity or evenness of any of the organism groups. This result is surprising given that an increase in habitat area often translates into a higher number of individuals and species as displayed in positive species-area relationships^28,35^. This positive relationship for plants and pollinators has been previously observed^46,47^, but there is also evidence showing that this assumption does not always hold^48,49^. In our landscapes, the proportion of semi-natural grasslands varied between 0.8 and 9.9%. It is possible this was too low, or that the variation in the area of semi-natural grasslands among landscapes was too small to detect effects on landscape-scale biodiversity.

Butterfly communities had a low evenness in all four landscape categories. This is most likely attributed to the dominance of *Aphantopus hyperantus* which had more than three times the number of individuals of the second most abundant species, *Coenonympha arcania*, and is also the most frequently observed species in the national Swedish butterfly monitoring programme^50^. Similarly, bumblebee communities were mostly dominated by one species, *Bombus pascuroum*, in all four landscape categories. As these patterns were observed in all four landscape categories, they were not driven by the area of linear infrastructure habitats in the landscape. Evenness of communities can be strongly affected by habitat area and connectivity^51,52^. One reason why we found no difference in evenness of butterfly and bumblebee communities between landscape categories might be that habitat area and connectivity can have opposite effects of evenness^7^, but we could not separate between these effects.

The evenness of plants responded differently to the area of road verges in the landscapes with and without power lines. Because the abundance of plants and flower-visiting insects were measured in different ways, the evenness of plants cannot be directly compared with the evenness of the flower-visiting insects. For plants, we measured the frequency of occurrence in 1 m^2^-plots, rather than estimating actual abundance. As a result, Shannon evenness values for plants were high in all landscape categories. However, our results still indicated that the presence of power line corridors in the landscape did not only have a positive effect on the number of plant species but also on their evenness.

Changes in landscape structure can induce a non-random loss of species which can generate phylogenetically clustered communities^53^. This is because closely related species often share similar traits (i.e. niche conservatism) which can result in similar responses to land-use change^54^. Based on this, we expected that an increase in habitat amount and landscape connectivity would translate into less phylogenetically clustered communities (i.e. higher phylogenetic diversity), as local diversity would be mostly driven by colonisations from a more diverse regional species pool^55^. However, we found no difference in phylogenetic diversity between landscape categories. Thus, our results indicate that if the landscapes are losing species^2,38^, the loss of plant and flower-visiting insect species is likely to be random and thereby not generating phylogenetically clustered communities. Given that all bumblebees are relatively closely related, the lack of relationship between the landscape features and their phylogenetic diversity is perhaps not so surprising even though bumblebee clades differ in important traits such as tongue length^56^. Further investigations on how species groups with different degree of relatedness are affected by changes in land use are needed to determine whether the evolutionary tree is in fact being pruned in such a way that ecosystems are less resilient to disturbances.

### Conclusions

It is important to determine whether novel grassland habitats, such as those along linear infrastructures, can complement or even replace traditionally managed semi-natural grasslands. However, very little is known about the contribution of these novel habitats for biodiversity at larger spatial scales^21^. Our results suggest that power line corridors can contribute to landscape-scale biodiversity at least of plants, whereas the effect of road verges is less clear.

Since there was no relationship between linear infrastructure habitats and the biodiversity of flower-visiting insects, future investigations should address whether linear infrastructure habitats are acting as sinks or sources for insect populations. It will also be important to investigate whether linear infrastructure habitats are affecting other pollinating groups such as solitary bees and moths. Estimating parameters such as population growth and mortality of generalist and specialist species within these habitats would increase our understanding on the mechanisms driving landscape biodiversity. Another important aspect to take into account is the amount of variation that can be explained by the local edaphic conditions and the past habitat area and connectivity of semi-natural grasslands in the landscapes. This can partly be done by using historical maps and aerial photographs, and it could reveal whether the landscapes with power line corridors are suffering from extinction debt and introducing this data into our analyses might also affect the reported results.

We showed that landscapes with high amount of linear infrastructure habitats held the same biodiversity as did landscapes with low amount of these habitats. Assessments of the value for conservation of linear infrastructure habitats in landscapes that have undergone complete loss of semi-natural habitat are needed as their value can be relative to the remaining amount of habitat.

## Methods

### Experimental design and landscape selection

**Figure 5:**
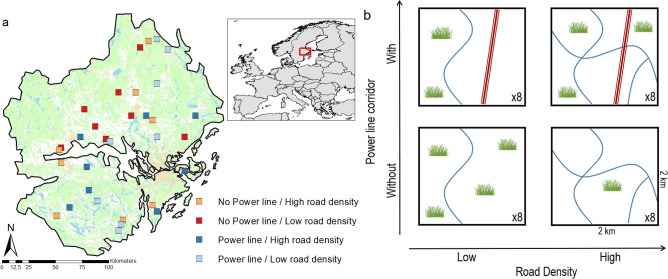
(**a**) Location of the 32 landscapes in Sweden and (**b**) study design. The grass illustration in the landscapes is a simplified representation of the varying areas of semi-natural grasslands within all 32 landscapes. (**a**) Was created with ArcMap 10.7: https://desktop.arcgis.com/en/arcmap/.

We established a study design that aimed to examine the effects of two types of linear infrastructure habitats, power line corridors and road verges of small and big roads, on the biodiversity of plants and flower-visiting insects at the landscape scale. To do this, we selected 32 landscapes of 4 km^2^ in area in Sweden (Fig. 5a) that fell into four different categories in order to achieve a crossed design: 16 of the landscapes included a power line corridor running through forest (minimum distance of power line corridor through forest = 1.0 km) and the other 16 did not have any power line corridor, 16 landscapes had a high road density, used as a proxy for road verge area, while the other 16 had a low road density (Fig. 5b). There was also a gradient in the area of semi-natural grasslands within each landscape category, independent of both the presence of power line corridors and road verge density. The landscapes were selected by first creating a 2 × 2 km grid over east-central Sweden and calculating the areas of forest and arable lands as well as the length of power-line corridors and public and private roads using the Swedish Terrain Map (GSD Geografiska Sverigedata), and the area of semi-natural grasslands using the TUVA database (http://www.jordbruksverket.se/tuva). The grasslands included in the TUVA database are of high nature value and consist of managed semi-natural pastures and meadows^57^. Based on this data we selected forest-dominated landscapes (45–81% forest) with the maximum possible contrasts in road density, presence vs. absence of power-line corridors, and a gradient in the area of semi-natural grasslands ranging between 0.8 and 9.9% (Supplementary Table S1). The selected landscapes were forest-dominated partly because Sweden is mostly dominated by forests, but most importantly because power line corridors only occur when crossing forested areas.

### Plant and flower-visiting insect surveys

In each of the 32 landscapes, we selected one grazed (or sometimes previously grazed) semi-natural grassland, one road verge along a larger paved road (minimum width = 1.5 m, max width = 5 m, average width = 2.8 m) and one along a smaller gravel road (minimum width = 1.5 m, maximum width = 5.5 m, average width = 2.9 m), and one power line corridor (minimum width = 28 m, maximum width = 95 m, average width = 39.9 m) in the 16 landscapes which include power lines. Power line corridors in this region in Sweden are managed by clearing young trees and tall shrubs every eight years. One of the corridors had been mowed earlier in the same year of the sampling (2016) and the oldest succession was in a corridor that had not been mowed for six years. The rest of the power line sites were mowed sometime between 2010 and 2016 with an average of three years since the last mowing. Given their relative simplicity to recognize in the field, we sampled plants, butterflies, burnet moths, and bumblebee communities in each of these habitats, within each of the 32 landscapes along a 200 m long transect in each habitat. This transect was then divided into four 50 m sections. Butterflies and burnet moths were inventoried along the transect with a “Pollard walk”^58^, i.e. the number of individuals of each species observed within five meters from the observer were noted (2.5 m at each side and five meters upwards and to the front). However, when performing the inventory in road verges that were narrower than 5 m, all individuals seen were noted. As burnet moths are day-flying and have similar requirements as some butterfly species they were treated as additional butterfly species and these two groups are hereinafter referred to as “butterflies”. Due to the difficulties of identifying specimens to species in the field, the species *Leptidea sinapis* and *L. juvernica* were pooled together as a single species. For bumblebees, all individuals present within 1 m at each side of the observer were caught and identified in the field. When the individual could not be identified, it was collected and identified later in the laboratory. However, no bumblebee queens were collected to minimize the impacts on the sampled populations. Butterflies and bumblebees were inventoried four times at each site between 1 June and 23 August 2016. These inventories were only conducted between 10:00 and 16:00 h, on days with no precipitation, and when the temperature was above 17 °C if the weather was sunny or above 20 °C if the weather was cloudy. The sequence of sites visited in the same day was randomized in order to mimimize any bias due to the diel activity pattern of the insects. The surveyors inventoried all habitats in order to avoid collector bias.

For the plant inventory, one quadratic sample plot was placed in the middle of each segment of the transect, i.e., amounting to four plots per transect. We recorded the presence or absence in the plot of all plant species from a pre-defined list including a total of 170 species that are commonly found in different types of grassland habitats. Hence, this list contained the absolute majority of the species present in each site. In addition to this, an inventory of plant indicator species which indicate long-term grassland management practices and/or adequate soil properties (e.g. absence of fertilizers) within the inventoried habitat was performed along the complete length of the transect in each habitat within all landscapes. The person performing the inventory was given a list of 55 species (adapted from Eneland 2017^57^) (Supplementary Table S5). Each site was visited once between 13th of July and 24th of August 2016.

### Land use classification

We estimated the areas of arable land, semi-natural pastures of high and low nature value (the latter being open areas not within the TUVA database and grassy uncultivated field borders between arable fields, power line corridors, road verges of public and private roads, water and urban land covers and railways within all landscapes in ArcMap 10.7^59^ using the same databases that were used for the landscape selection (Supplementary Table S1). The area of power line corridors in the 16 landscapes was calculated by estimating the mean width of the visited corridors (39.9 m). Only the fragments of power line corridors that crossed forest cover were included in the calculations because only these areas are managed in such a way that they turn out to resemble semi-natural pastures. The area of road verges was calculated in a similar manner. A mean width was calculated for the visited public (5.6 m) and private roads (5.9 m) in the landscapes. These values were then used to calculate the area of road verges in the landscapes by multiplying them with the total road length in each landscape.

### Analyses

In the analyses presented here, we only used data from semi-natural grasslands and road verges. The data collected in the power line corridors was not used in the analysis in order to avoid the sampling bias caused by the lack of power line corridors in half of the landscapes. The analyses were performed in R^60^. We modelled three measures of biodiversity at the landscape scale (ϒ diversity): species richness, evenness and phylogenetic diversity. For the species richness as a response variable, we pooled all the species found in the grassland habitats in each landscape excluding the species found in the power line corridors (i.e. only species found in road verges of small and big roads and in semi-natural grasslands were used for the analysis). We did this separately for butterflies, bumblebees, plants and indicator plant species. Species richness of butterflies, bumblebees, plants and indicator plant species were modelled separately with a general linear model as an additive effect of the amount of semi-natural grasslands, the presence/absence of power line corridors and the road verge density (high or low) in the landscape. For the species richness of indicator plant species we ran separate generalized linear models (GLM) with Poisson distribution instead of general linear models. Interactions between all of the variables were also tested in separate models and the lowest AIC was used for model selection. When the ΔAIC was < 2 between two or more models, the model with the lowest AIC that included one or more interactions was selected. Additionally, we performed a non-metric multidimensional scaling (NMDS) ordination in order to investigate if there were differences in the identities of species present in each landscape category.

To investigate whether linear infrastructure habitats have an effect on the abundance distributions of butterflies, bumblebees and plant species in the landscape (but not for indicator plant species, for which we only had presence/absence data) we calculated evenness separately for each group in every landscape using Shannon’s evenness index (E_Shannon_), which uses the Shannon diversity index but without the effect of richness. E_Shannon_ is given by:

$${\text{E}}_{{{\text{Shannon}}}} = - \sum p_{i} \ln p_{i} /\ln \left( {\text{S}} \right)$$

where *p*_*i*_ is the *i*th element of the vector of relative abundances *p* and S is the total number of species. E_Shannon_ takes values from zero to one, where one represents a situation in which all species in the community are present in equal proportions and zero represents the opposite. Then, we modelled evenness as a response to the amount of grasslands, the presence or absence of power line corridors and the density of road verges in the landscape. Additionally, we created diversity profiles for butterflies, bumblebees and all plants in each landscape category using the *vegan* package in R. This was done because restricting the evenness analysis to a single index can be misleading due to the different weights given by different indices to rare and common species, which can affect the relationship between evenness and land use^7^. Diversity profiles enable comparison between different diversity indices by using the effective number of species (Hill numbers) in order to characterize the diversities of different communities.

To calculate the phylogenetic diversity of each organism group, we created a phylogenetic tree for all the species found within the 32 landscapes (not including the species found in the power line corridors). We did this by downloading a phylogenetic tree from the Open Tree of Life (OTL: https://tree.opentreeoflife.org) using the *rotl* package in R. Two plant species (*Rosa dumalis* and *Potentilla tabernaemontani)* were removed because they were missing from the most recent taxonomy [access date: 08-01-2020]. We estimated the branch length for the tree containing all species using the *ape* package in R. This package uses Grafen’s method to calculate branch length; it sets branch lengths to a length equal to the number of descendant tips minus one. Then, Faith’s Phylogenetic Diversity Index was calculated for each landscape using the previously calculated branch lengths using *Picante* package in R. Because phylogenetic diversity is often correlated to species richness, it can prove difficult to determine if differences in phylogenetic diversity are real or due to differences in species richness. Thus, we performed 1000 tree randomizations to calculate the standardized effect size of phylogenetic diversity for each landscape. Finally, the standardized effect size of phylogenetic diversity was modelled as a response to the same explanatory variables as with species richness and evenness also using a general linear model.

## Supplementary information

Supplementary Information.

## Data Availability

The data supporting the findings of this publication are available in Figshare at 10.6084/m9.figshare.13286141.

## References

[1] Bergman K-O, Dániel-Ferreira J, Milberg P, Öckinger E, Westerberg L (2018). Butterflies in Swedish grasslands benefit from forest and respond to landscape composition at different spatial scales. Landsc. Ecol..

[2] Cousins SAO, Auffret AG, Lindgren J, Tränk L (2015). Regional-scale land-cover change during the 20th century and its consequences for biodiversity. Ambio.

[3] Eriksson O, Cousins SAO, Bruun HH (2002). Land-use history and fragmentation of traditionally managed grasslands in scandinavia. J. Veg. Sci..

[4] Tyler T (2020). Recent changes in the frequency of plant species and vegetation types in Scania, S Sweden, compared to changes during the twentieth century. Biodivers. Conserv..

[5] Thomas JA (2016). Butterfly communities under threat. Science.

[6] Bommarco R, Lundin O, Smith HG, Rundlöf M (2012). Drastic historic shifts in bumble-bee community composition in Sweden. Proc. R. Soc. B.

[7] Marini L (2014). Contrasting effects of habitat area and connectivity on evenness of pollinator communities. Ecography.

[8] Ferreira PA, Boscolo D, Viana BF (2013). What do we know about the effects of landscape changes on plant–pollinator interaction networks?. Ecol. Indic..

[9] Larsen TH, Williams NM, Kremen C (2005). Extinction order and altered community structure rapidly disrupt ecosystem functioning: altered community structure disrupts function. Ecol. Lett..

[10] Vanneste T (2020). Plant diversity in hedgerows and road verges across Europe. J. Appl. Ecol..

[11] Phillips, B. B. *et al.* Enhancing road verges to aid pollinator conservation: a review. *Biol. Conserv.* **250**, 108687. 10.1016/j.biocon.2020.108687 (2020).

[12] Berg Å, Bergman K-O, Wissman J, Żmihorski M, Öckinger E (2016). Power-line corridors as source habitat for butterflies in forest landscapes. Biol. Conserv..

[13] Lundholm JT, Richardson PJ (2010). MINI-REVIEW: habitat analogues for reconciliation ecology in urban and industrial environments. J. Appl. Ecol..

[14] Cranmer L, McCollin D, Ollerton J (2012). Landscape structure influences pollinator movements and directly affects plant reproductive success. Oikos.

[15] Van Geert A, Van Rossum F, Triest L (2010). Do linear landscape elements in farmland act as biological corridors for pollen dispersal? Linear landscape elements as corridors. J. Ecol..

[16] Lázaro-Lobo A, Ervin GN (2019). A global examination on the differential impacts of roadsides on native vs. exotic and weedy plant species. Glob. Ecol. Conserv..

[17] Dubé C, Pellerin S, Poulin M (2011). Do power line rights-of-way facilitate the spread of non-peatland and invasive plants in bogs and fens?. Botany.

[18] Fahrig, L. & Rytwinski, T. Effects of Roads on Animal Abundance: an Empirical Review and Synthesis. *Ecol. Soc.* **14**(1): 21. http://www.ecologyandsociety.org/vol14/iss1/art21/ (2009).

[19] Benítez-López A, Alkemade R, Verweij PA (2010). The impacts of roads and other infrastructure on mammal and bird populations: a meta-analysis. Biol. Conserv..

[20] Keilsohn W, Narango DL, Tallamy DW (2018). Roadside habitat impacts insect traffic mortality. J. Insect Conserv..

[21] Gardiner MM, Riley CB, Bommarco R, Öckinger E (2018). Rights-of-way: a potential conservation resource. Front. Ecol. Environ..

[22] Phillips BB, Gaston KJ, Bullock JM, Osborne JL (2019). Road verges support pollinators in agricultural landscapes, but are diminished by heavy traffic and summer cutting. J. Appl. Ecol..

[23] Wagner DL, Metzler KJ, Frye H (2019). Importance of transmission line corridors for conservation of native bees and other wildlife. Biol. Conserv..

[24] Wojcik VA, Buchmann S (2012). Pollinator conservation and management on electrical transmission and roadside rights-of-way: a review. J. Pollinat. Ecol..

[25] Stenmark, M. Infrastrukturens gräs-och buskmarker. Hur stora arealer gräs och buskmarker finns i anslutning till transportinfrastruktur och bidrar dessa till miljömålsarbetet? *Infrastrukturens gräs-och buskmarker.* Jordbruksverket Rapport 2012:36 (2012).

[26] Jeusset A (2016). Can linear transportation infrastructure verges constitute a habitat and/or a corridor for biodiversity in temperate landscapes? A systematic review protocol. Environ. Evid..

[27] Crist TO, Veech JA, Gering JC, Summerville KS (2003). Partitioning species diversity across landscapes and regions: a hierarchical analysis of α, β, and γ diversity. Am. Nat..

[28] With KA (2016). Are landscapes more than the sum of their patches?. Landsc. Ecol..

[29] Cornell HV, Harrison SP (2014). What are species pools and when are they important?. Annu. Rev. Ecol. Evol. Syst..

[30] Cornell HV, Lawton JH (1992). Species interactions, local and regional processes, and limits to the richness of ecological communities: a theoretical perspective. J. Anim. Ecol..

[31] Steinert M, Moe SR, Sydenham MAK, Eldegard K (2018). Different cutting regimes improve species and functional diversity of insect-pollinated plants in power-line clearings. Ecosphere.

[32] Gagic V (2015). Functional identity and diversity of animals predict ecosystem functioning better than species-based indices. Proc. R. Soc. B Biol. Sci..

[33] Chao A, Chiu C-H, Jost L (2014). Unifying species diversity, phylogenetic diversity, functional diversity, and related similarity and differentiation measures through hill numbers. Annu. Rev. Ecol. Evol. Syst..

[34] Vellend, M., Cornwell, W. K., Magnuson-Ford, K. & Mooers, A. O. Measuring phylogenetic biodiversity. In *Biological Diversity: Frontiers in Measurement and Assessment* 194–207 (Oxford University Press, 2011).

[35] Rosenzweig ML (1995). Species Diversity in Space and Time.

[36] Fahrig L (2013). Rethinking patch size and isolation effects: the habitat amount hypothesis. J. Biogeogr..

[37] Hill B, Bartomeus I (2016). The potential of electricity transmission corridors in forested areas as bumblebee habitat. R. Soc. Open Sci..

[38] Kuussaari M (2009). Extinction debt: a challenge for biodiversity conservation. Trends Ecol. Evol..

[39] Krauss J (2010). Habitat fragmentation causes immediate and time-delayed biodiversity loss at different trophic levels: Immediate and time-delayed biodiversity loss. Ecol. Lett..

[40] Grusell, E. & Miliander, S. *Fältmanual för skötsel av kraftledningsgatans biotoper*. https://www.svk.se/contentassets/2f77f2d04b7b451495013f4de5fa7409/bilaga-5-faltmanual-for-skotsel-av-kraftledningsgatans-biotoper.pdf (2011).

[41] Zeiter M, Stampfli A, Newbery DM (2006). Recruitment limitation constrains local species richness and productivity in dry grassland. Ecology.

[42] Chaudron C, Chauvel B, Isselin-Nondedeu F (2016). Effects of late mowing on plant species richness and seed rain in road verges and adjacent arable fields. Agric. Ecosyst. Environ..

[43] Angold PG (1997). The impact of a road upon adjacent heathland vegetation: effects on plant species composition. J. Appl. Ecol..

[44] Watmough SA, Rabinowitz T, Baker S (2017). The impact of pollutants from a major northern highway on an adjacent hardwood forest. Sci. Total Environ..

[45] Andersson P, Koffman A, Sjödin NE, Johansson V (2017). Roads may act as barriers to flying insects: species composition of bees and wasps differs on two sides of a large highway. Nat. Conserv..

[46] Öckinger E, Smith HG (2006). Semi-natural grasslands as population sources for pollinating insects in agricultural landscapes: population sources for pollinators. J. Appl. Ecol..

[47] Krauss J, Klein A-M, Steffan-Dewenter I, Tscharntke T (2004). Effects of habitat area, isolation, and landscape diversity on plant species richness of calcareous grasslands. Biodivers. Conserv..

[48] Thiele J, Kellner S, Buchholz S, Schirmel J (2018). Connectivity or area: what drives plant species richness in habitat corridors?. Landsc. Ecol..

[49] Lampinen J, Heikkinen RK, Manninen P, Ryttäri T, Kuussaari M (2018). Importance of local habitat conditions and past and present habitat connectivity for the species richness of grassland plants and butterflies in power line clearings. Biodivers. Conserv..

[50] Pettersson, L. B., Arnberg, H. & Mellbrand, K. *Svensk Dagfjärilsövervakning Årsrapport 2018*. (2018).

[51] Orrock JL, Curler GR, Danielson BJ, Coyle DR (2011). Large-scale experimental landscapes reveal distinctive effects of patch shape and connectivity on arthropod communities. Landsc. Ecol..

[52] Clough Y (2014). Density of insect-pollinated grassland plants decreases with increasing surrounding land-use intensity. Ecol. Lett..

[53] Grab H (2019). Agriculturally dominated landscapes reduce bee phylogenetic diversity and pollination services. Science.

[54] Williams NM (2010). Ecological and life-history traits predict bee species responses to environmental disturbances. Biol. Conserv..

[55] Helmus MR, Ives AR (2012). Phylogenetic diversity—area curves. Ecology.

[56] Cameron SA, Hines HM, Williams PH (2007). A comprehensive phylogeny of the bumble bees (Bombus). Biol. J. Linn. Soc..

[57] Eneland, A. Ängs- och betesmarksinventeringen. Metodik för inventering från och med 2016. Jordbruksverket Rapport 2017:9 (2017).

[58] Pollard E (1977). A method for assessing changes in the abundance of butterflies. Biol. Conserv..

[59] ESRI. ArcGIS Desktop: Release 10. Redlands, CA: Environmental Systems Research Institute (2018). https://desktop.arcgis.com/en/arcmap/.

[60] R Core Team. *R: A language and environment for statistical computing. R Foundation for Statistical Computing*. (2019).

