# Author Correction: Linear infrastructure habitats increase landscape-scale diversity of plants but not of flower-visiting insects

**DOI:** 10.1038/s41598-021-86223-0

**Published:** 2021-03-22

**Authors:** Juliana Dániel-Ferreira, Riccardo Bommarco, Jörgen Wissman, Erik Öckinger

**Affiliations:** 1 Department of Ecology, Swedish University of Agricultural Sciences, Box 7044, 75007 Uppsala, Sweden; 2 Swedish Biodiversity Centre, CBM, Swedish University of Agricultural Sciences, Box 7016, 75007 Uppsala, Sweden

Correction to: *Scientific Reports* 10.1038/s41598-020-78090-y, published online 07 December 2020

The original version of this Article contained a typographical error in the Abstract.

“Habitats along linear infrastructure, such as roads and electrical transmission lines, can have high local biodiversity. To determine whether these habitats also contribute to landscape-scale biodiversity, we estimated species richness, evenness and phylogenetic diversity of plant, butterfly and bumblebee communities in 32 km^2^ landscapes with or without power line corridors, and with contrasting areas of road verges. Landscapes with power line corridors had on average six more plant species than landscapes without power lines, but there was no such effect for butterflies and bumblebees. Plant communities displayed considerable evenness in species abundances both in landscapes with and without power lines and high and low road verge densities. We hypothesize that the higher number of plant species in landscapes with power line corridors is due to these landscapes having a higher extinction debt than the landscapes without power line corridors, such that plant diversity is declining slower in landscapes with power lines. This calls for targeted conservation actions in semi-natural grasslands within landscapes with power line corridors to maintain biodiversity and prevent imminent population extinctions.”

now reads:

“Habitats along linear infrastructure, such as roads and electrical transmission lines, can have high local biodiversity. To determine whether these habitats also contribute to landscape-scale biodiversity, we estimated species richness, evenness and phylogenetic diversity of plant, butterfly and bumblebee communities in 32 4 km^2^ landscapes with or without power line corridors, and with contrasting areas of road verges. Landscapes with power line corridors had on average six more plant species than landscapes without power lines, but there was no such effect for butterflies and bumblebees. Plant communities displayed considerable evenness in species abundances both in landscapes with and without power lines and high and low road verge densities. We hypothesize that the higher number of plant species in landscapes with power line corridors is due to these landscapes having a higher extinction debt than the landscapes without power line corridors, such that plant diversity is declining slower in landscapes with power lines. This calls for targeted conservation actions in semi-natural grasslands within landscapes with power line corridors to maintain biodiversity and prevent imminent population extinctions.”

This error has now been corrected in the PDF and HTML versions of the Article.

