# Supplementary material for: Linear infrastructure habitats increase landscape-scale diversity of plants but not of flower-visiting insects
Source: Sci Rep. 2020 Dec 7;10:21374. doi: 10.1038/s41598-020-78090-y (PMC7721902; doi:10.1038/s41598-020-78090-y)
Supplement: Supplementary file 1 — Supplementary Information. [file 41598_2020_78090_MOESM1_ESM.pdf]

Linear infrastructure habitats increase landscape-scale diversity of plants but not of flower-visiting insects

Juliana Dániel-Ferreira, Riccardo Bommarco, Jörgen Wissman, Erik Öckinger

Supplementary information

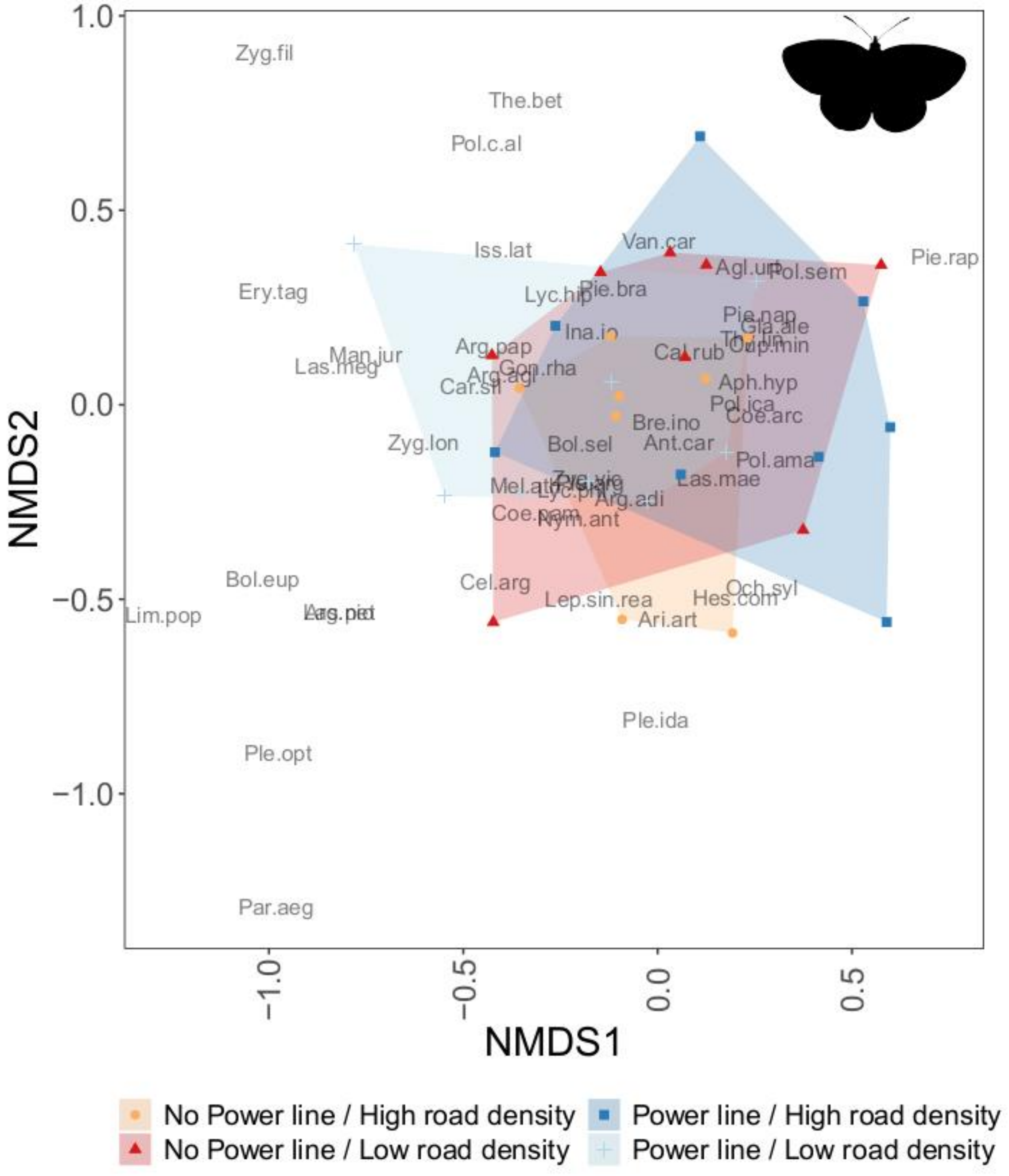

Figure S1. Non-metric multidimensional scaling (NMDS) analysis for butterflies (stress=0.23; non-metric fit R^2= 0.946; linear fit, R^2= 0.721) in the four landscape categories. There are not evident differences in the species compositions between landscape categories. See Table S2 for the corresponding names to the abbreviations. The silhouette image was available under Public Domain license at PhyloPic (http://phylopic.org).

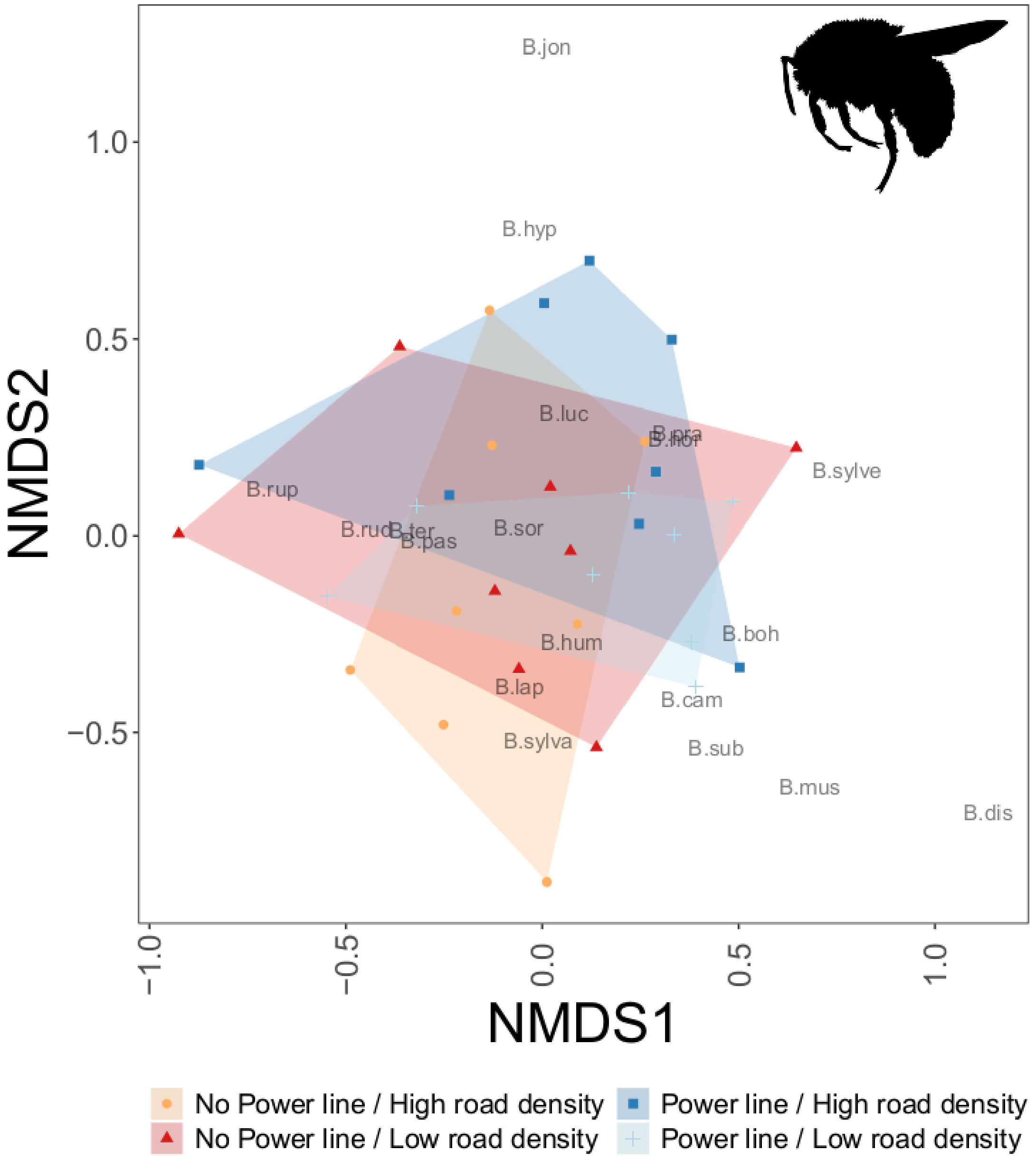

Figure S2. Non-metric multidimensional scaling (NMDS) analysis for bumblebees (stress=0.24; non-metric fit R^2= 0.943; linear fit, R^2= 0.699) in the four landscape categories. There are not evident differences in the species compositions between landscape categories. The silhouette image was available under Public Domain license at PhyloPic (http://phylopic.org).

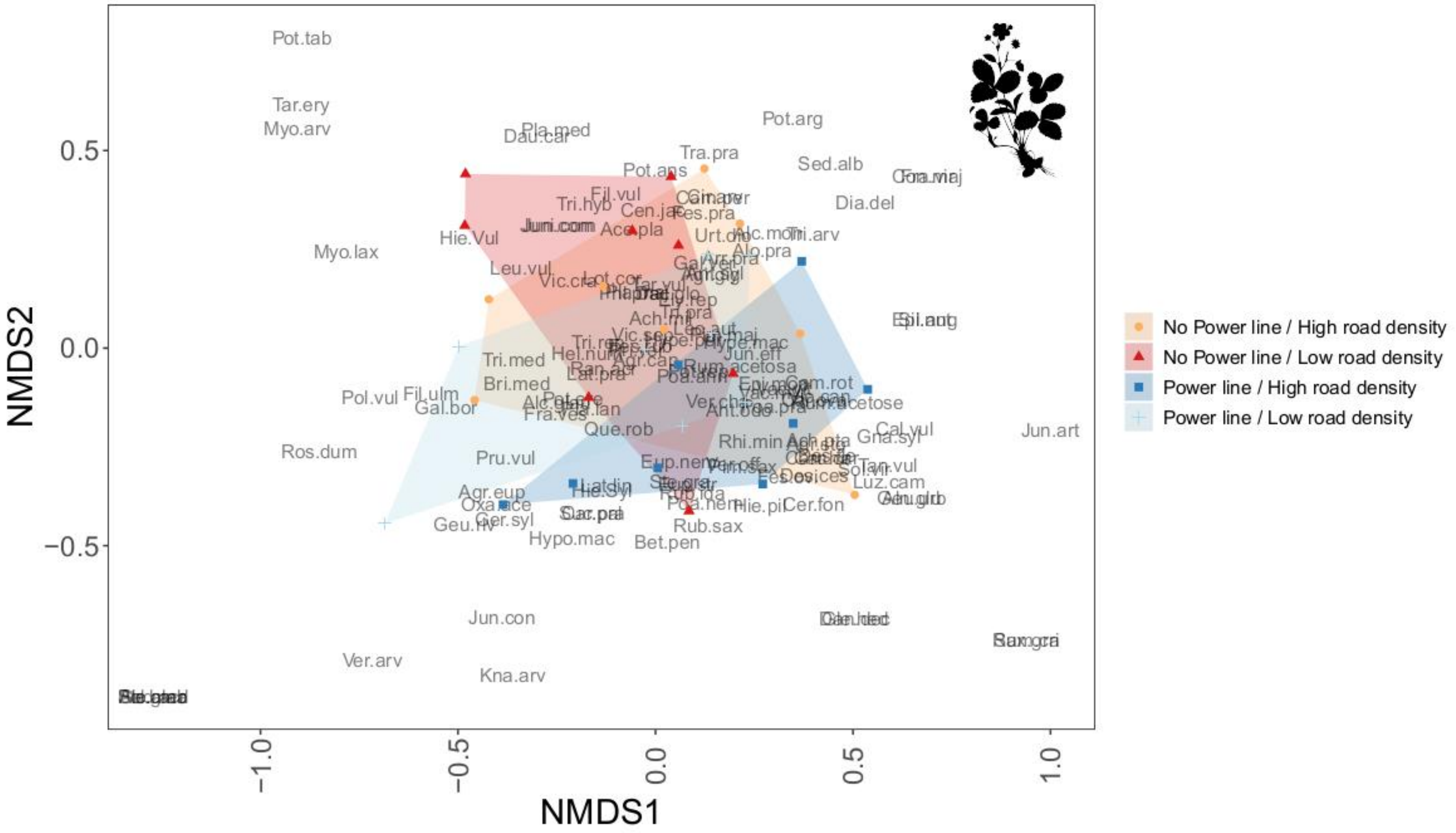
NMDS2
NMDS1
0.5
0.0
−0.5
−1.0
−0.5
0.0
0.5
1.0
No Power line / High road density
No Power line / Low road density
Power line / High road density
Power line / Low road density
Pot.tab
Tar.ery
Myo.arv
Myo.lax
Pot.arg
Sed.alb
Dia.del
Tra.pra
Pot.ans
Hie.Vul
Leu.vul
Tri.med
Bri.med
Pol.vul
Gal.bor
Ros.dum
Pru.vul
Agr.eup
Geu.riv
Ger.syl
Hypo.mac
Bet.pen
Rub.sax
Cer.fon
Luz.cam
Cal.vul
Jun.art
Que.rob
Rhi.min
Jun.con
Ver.arv
Kna.arv

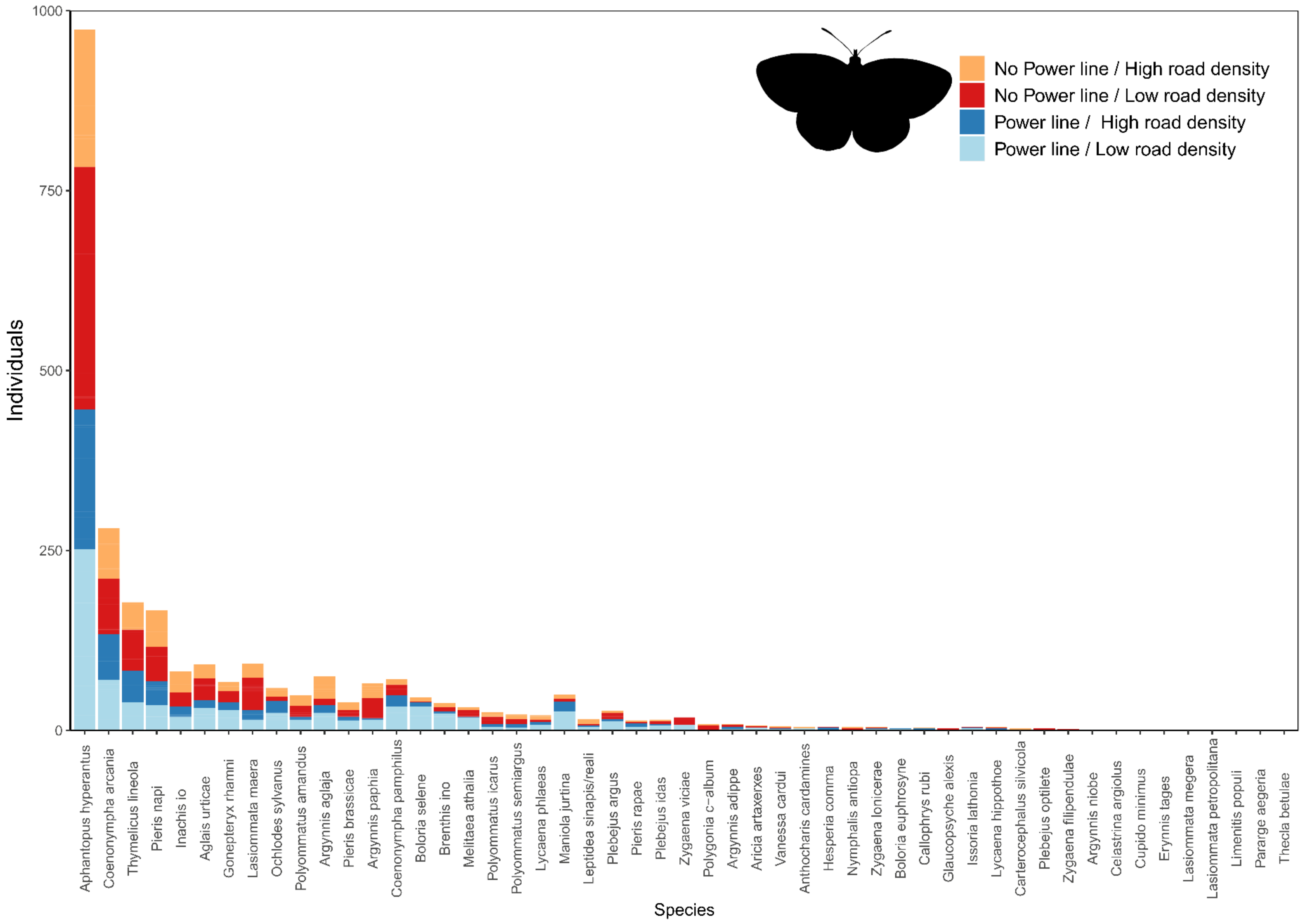

Figure S4. Abundance distribution of butterflies in the four landscape categories. Each colour represents a landscape category. The silhouette image was available under Public Domain license at PhyloPic (http://phylopic.org).

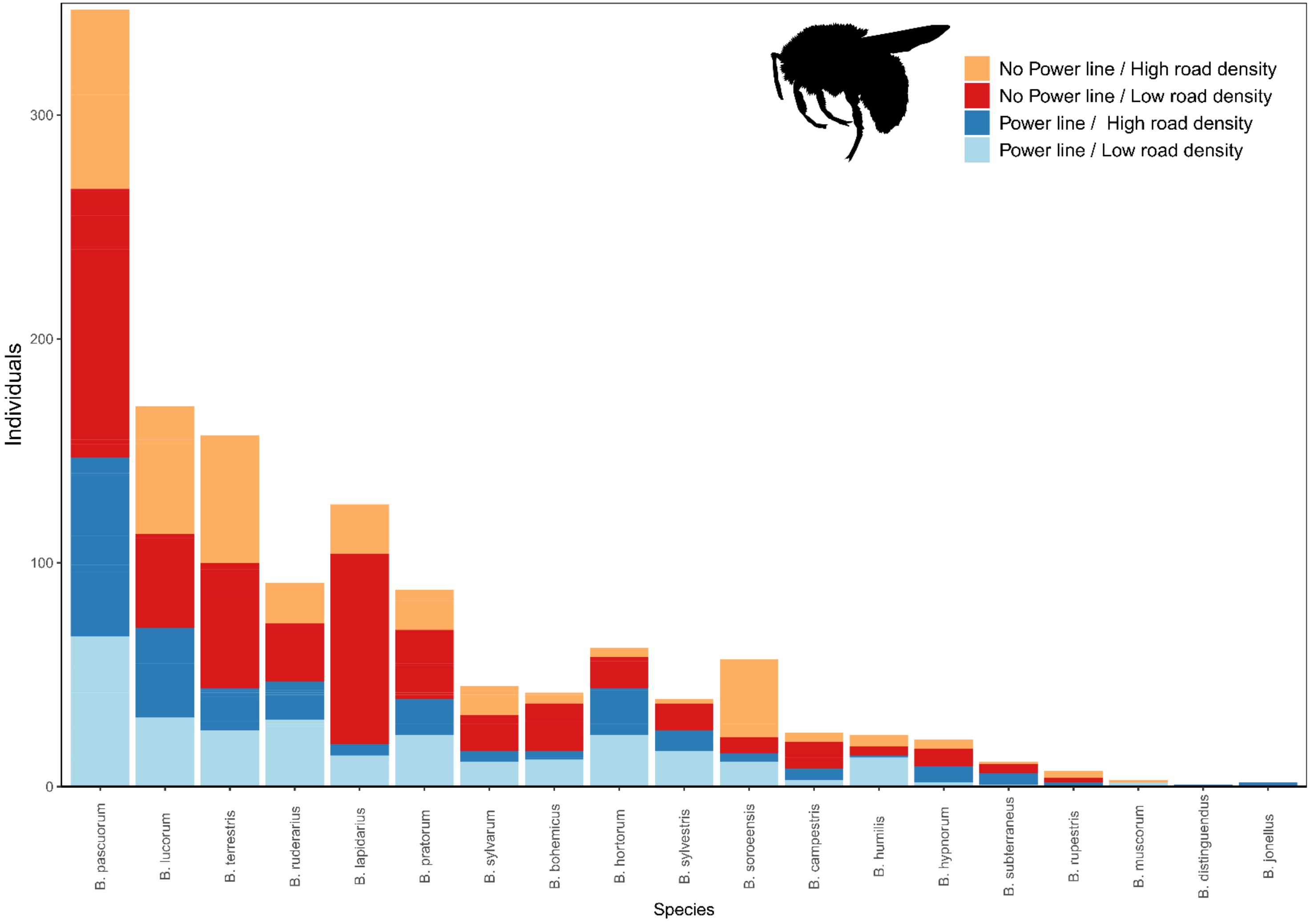

Figure S5. Abundance distribution of bumblebees in the four landscape categories. Each colour represents a landscape category. The silhouette image was available under Public Domain license at PhyloPic (http://phylopic.org).

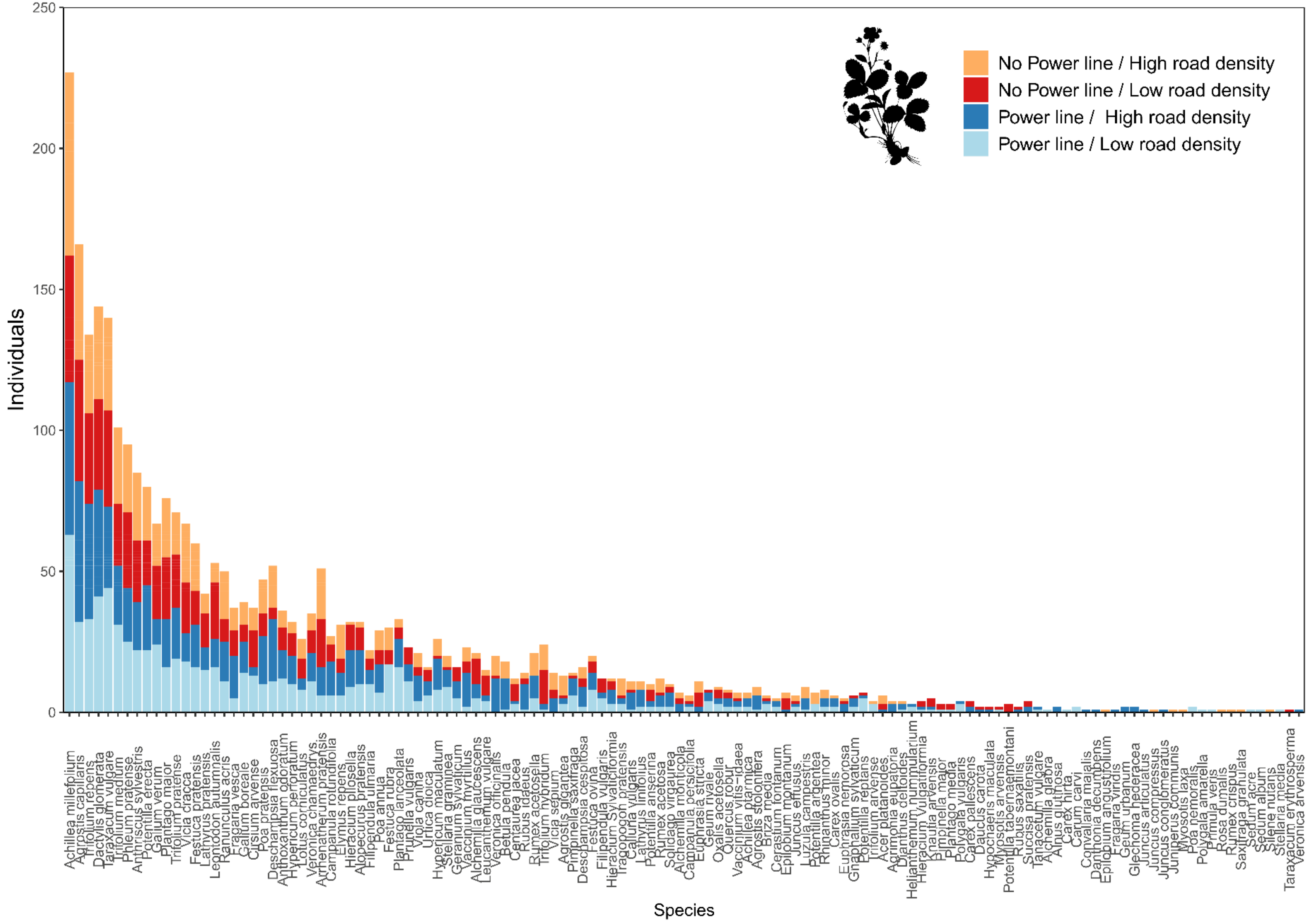

Figure S6. Abundance distribution of plants in the four landscape categories. Each colour represents a landscape category. The silhouette image was available under Public Domain license at PhyloPic (http://phylopic.org).

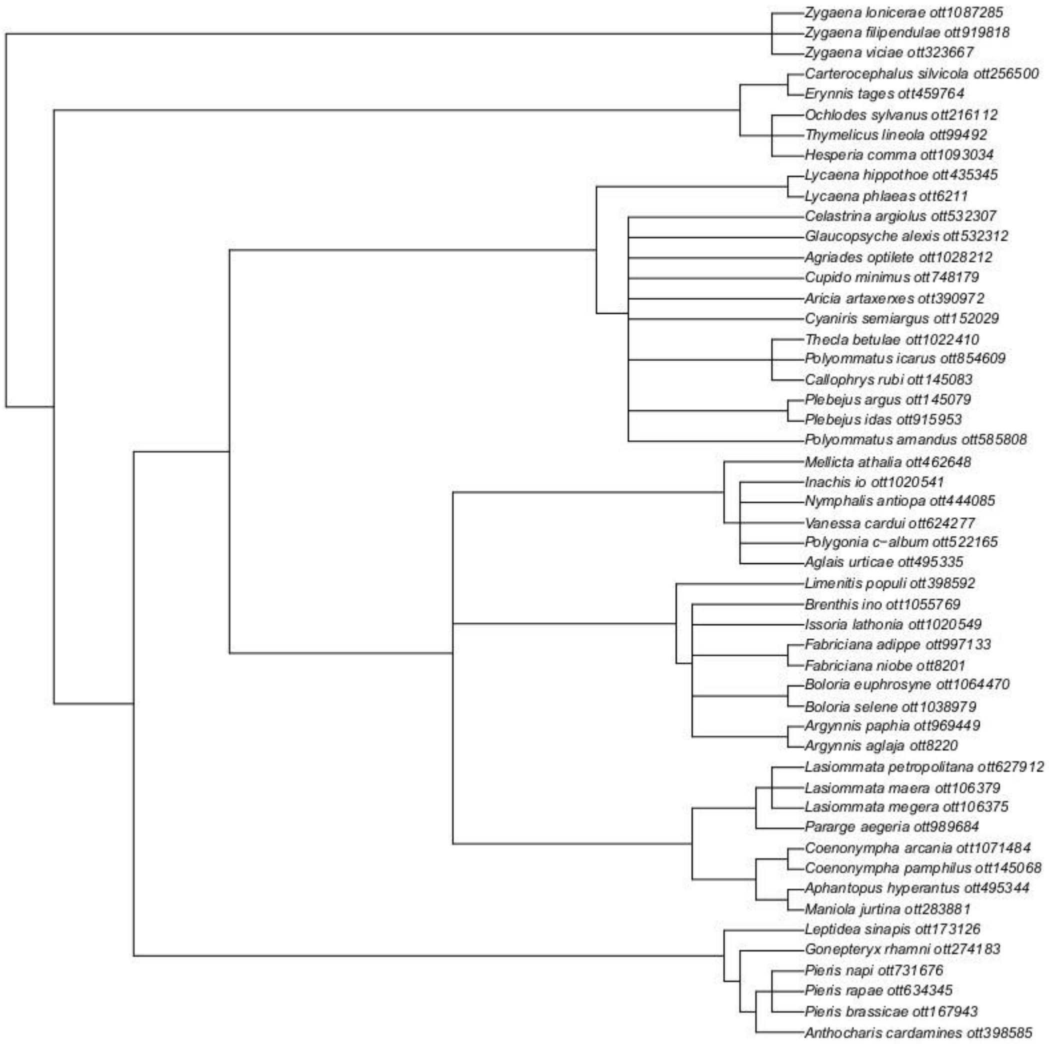

Figure S7. Synthetic tree for the butterfly species recorded in the study. Each species is accompanied by an Open Tree Taxonomy Identifier (OTT id). The tree was created using the *rotl* package in R (refer to main text for more details).

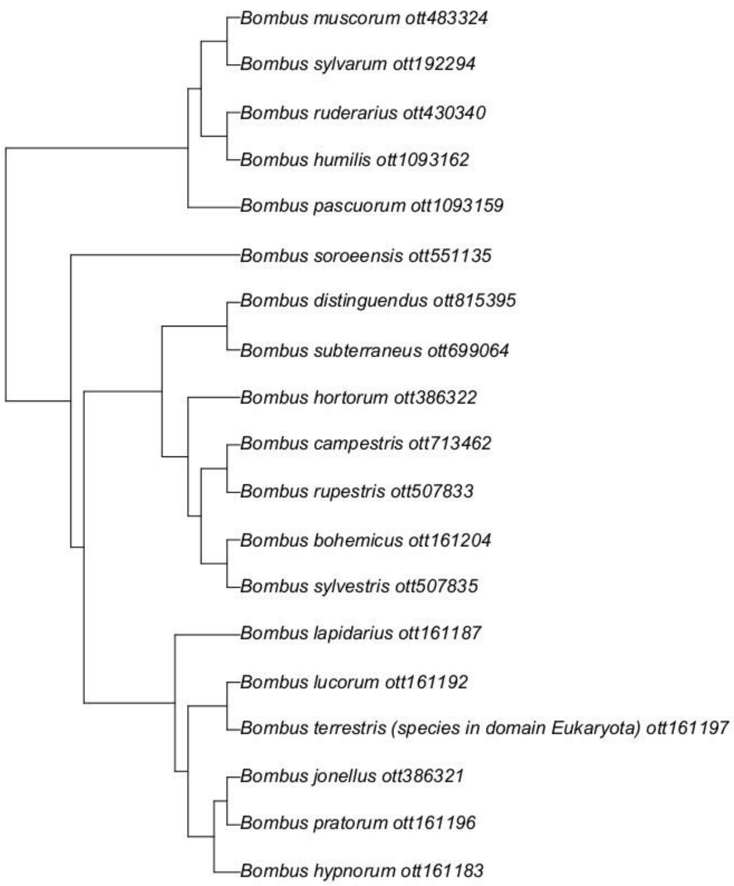

Figure S8. Synthetic tree for the bumblebee species recorded in the study. Each species is accompanied by an Open Tree Taxonomy Identifier (OTT id). The tree was created using the *rotl* package in R (refer to main text for more details).

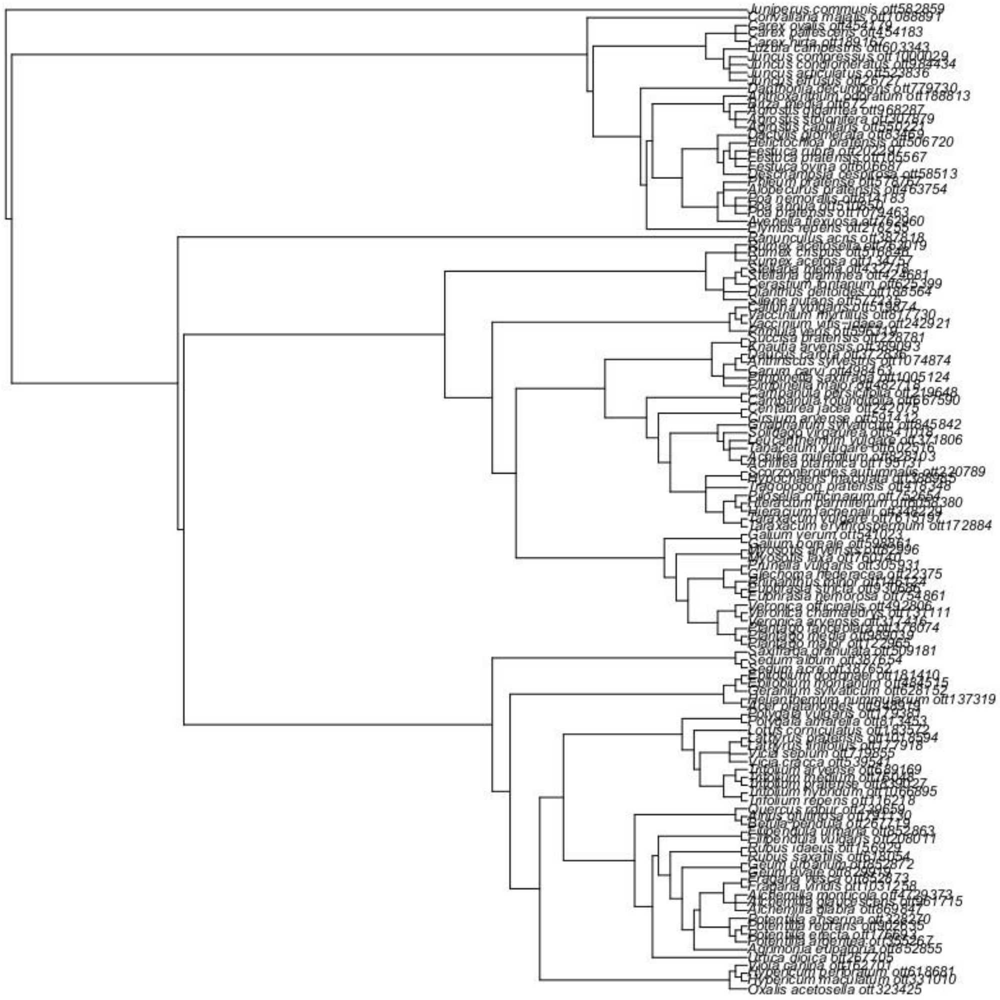

Figure S9. Synthetic tree for the vascular plant species recorded in the study. Each species is accompanied by an Open Tree Taxonomy Identifier (OTT id). The tree was created using the *rotl* package in R (refer to main text for more details).

Table S1. **Percentage of measured land-use types in the studied landscapes.** Landscapes are organized according to the percentage of semi-natural grasslands in the landscape, from lowest to highest. Landscape abbreviations: NoPL.LRD= absence of power line corridor and low road density; NoPL.HRD= presence of power line corridor and high road density; PL.LRD= presence of power line corridor and low road density; PL.HRD= presence of power line corridor and high road density.

Landscape	Category	Semi-natural grasslands	Power lines	Railway	Roads (small)	Public roads (big)	Grassy uncultivated field borders	Forest	Arable Land	Open area	Urban cover	Water
7073	NoPL.LRD	0.8	0.0	0.0	0.8	0.5	0.1	58.8	33.2	5.8	0.0	0.0
5683	NoPL.LRD	1.0	0.0	0.0	0.7	0.2	0.1	76.0	15.6	6.4	0.1	0.0
2844	PL.HRD	1.1	0.3	0.0	1.1	0.9	0.1	69.9	21.2	3.9	0.0	1.6
2333	PL.LRD	1.2	1.5	1.5	0.6	0.2	0.0	69.6	10.8	6.4	0.0	8.2
5561	PL.LRD	1.2	1.6	0.0	0.2	0.2	0.2	61.6	29.7	5.3	0.0	0.0
2156	NoPL.HRD	1.3	0.0	0.0	1.2	0.9	0.2	77.2	12.0	4.6	2.7	0.0
3897	PL.HRD	1.3	1.7	0.0	1.1	1.9	0.0	68.2	5.2	10.2	0.5	10.0
9931	NoPL.LRD	1.3	0.0	0.0	0.6	0.1	0.2	79.8	13.4	4.6	0.0	0.0
1851	PL.HRD	1.3	0.6	0.4	1.5	3.2	0.2	47.5	23.0	15.9	6.3	0.0
1037	PL.LRD	1.7	1.0	0.0	0.6	0.0	0.0	79.8	11.2	5.6	0.0	0.0
7411	NoPL.LRD	1.8	0.0	0.0	0.5	0.2	0.2	64.2	28.2	4.9	0.0	0.0
8768	PL.LRD	1.9	5.5	0.0	0.6	0.0	0.3	61.7	26.4	3.6	0.0	0.0
5798	PL.HRD	2.3	0.4	0.0	1.0	1.1	0.3	49.1	42.6	3.4	0.0	0.0
5834	PL.LRD	2.3	1.1	0.0	0.7	0.1	0.2	66.9	22.8	5.9	0.0	0.0
10099	NoPL.HRD	2.4	0.0	0.0	1.3	0.7	0.0	71.3	1.1	21.9	0.0	1.4
5847	NoPL.LRD	2.5	0.0	0.0	0.5	0.2	0.0	81.2	2.1	4.9	0.0	8.6
8329	NoPL.LRD	2.6	0.0	0.0	0.7	0.1	0.2	60.9	26.1	9.4	0.0	0.0
7103	PL.HRD	2.6	3.3	0.0	1.2	0.6	0.5	50.5	32.4	8.3	0.0	0.6
7080	PL.HRD	2.7	1.6	0.0	1.0	1.4	0.5	51.1	31.6	10.2	0.0	0.0
6437	NoPL.LRD	3.3	0.0	0.0	0.6	0.2	0.3	62.7	25.0	8.0	0.0	0.0
2014	PL.HRD	3.8	1.6	0.8	1.3	0.9	0.2	59.2	22.4	9.5	0.0	0.3
1605	NoPL.HRD	4.0	0.0	0.0	1.0	1.0	0.0	68.5	14.2	7.2	0.0	4.0
1278	PL.LRD	4.2	2,3	0.5	0.5	0.2	0.1	79.1	10.3	2.2	0.6	0.0
4091	PL.HRD	4.4	2,4	0.0	1.2	0.9	0.0	45.2	34.6	10.7	0.0	0.6
8337	NoPL.HRD	4.4	0.0	0.0	1.4	1.0	0.2	53.0	32.0	8.0	0.0	0.0
5041	NoPL.HRD	4.6	0.0	0.0	1.1	0.7	0.1	49.9	26.2	9.8	0.0	7.7
4319	NoPL.HRD	5.4	0.0	0.0	1.1	1.1	0.2	47.2	34.7	10.1	0.1	0.0
5166	NoPL.LRD	5.6	0.0	0.0	0.5	0.2	0.3	56.0	30.8	6.7	0.0	0.0
6836	NoPL.HRD	6.8	0.0	0.0	1.5	0.6	0.1	58.4	18.5	14.1	0.0	0.0
10156	PL.LRD	6.9	4.5	0.0	0.7	0.0	0.1	79.2	2.7	4.7	0.0	1.1
9889	PL.LRD	9.0	1.1	0.0	0.1	0.4	0.2	57.8	13.3	17.8	0.0	0.3
1450	NoPL.HRD	9.9	0.0	0.0	0.9	1.2	0.1	66.1	13.8	6.7	0.0	1.4

Table S2. List of recorded butterfly species and their respective abbreviations.

Species	Abbreviation	Species	Abbreviation
Aglais urticae	*Agl.urt*	*Leptidea sinapis / reali*	*Lep.sin/rea*
Anthocharis cardamines	*Ant.car*	*Limenitis populi*	*Lim.pop*
Aphantopus hyperantus	*Aph.hyp*	*Lycaena hippothoe*	*Lyc.hip*
Argynnis adippe	*Arg.adi*	*Lycaena phlaeas*	*Lyc.phl*
Argynnis aglaja	*Arg.agl*	*Maniola jurtina*	*Man.jur*
Argynnis niobe	*Arg.nio*	*Melitaea athalia*	*Mel.ath*
Argynnis paphia	*Arg.pap*	*Nymphalis antiopa*	*Nym.ant*
Aricia artaxerxes	*Ari.art*	*Ochlodes Sylvanus*	*Och.syl*
Boloria euphrosyne	*Bol.eup*	*Pararge aegeria*	*Par.aeg*
Boloria selene	*Bol.sel*	*Pieris brassicae*	*Pie.bra*
Brenthis ino	*Bre.ino*	*Pieris napi*	*Pie.nap*
Callophrys rubi	*Cal.rub*	*Pieris rapae*	*Pie.rap*
Carterocephalus silvicola	*Car.sil*	*Plebejus argus*	*Ple.arg*
Celastrina argiolus	*Cel.arg*	*Plebejus idas*	*Ple.ida*
Coenonympha arcania	*Coe.arc*	*Plebejus optiliete*	*Ple.opt*
Coenonympha pamphilus	*Coe.pam*	*Polygonia c-album*	*Pol.c-al*
Cupido minimus	*Cup.min*	*Polyommatus amandus*	*Pol.ama*
Erynnis tages	*Ery.tag*	*Polyommatus icarus*	*Pol.ica*
Glaucopsyche alexis	*Gla.ale*	*Polyommatus semiargus*	*Pol.sem*
Gonepterys rhamni	*Gon.rha*	*Thecla betulae*	*The.bet*
Hesperia comma	*Hes.com*	*Thymelicus lineola*	*Thy.lin*
Inachis io	*Ina.io*	*Vanessa cardui*	*Van.car*
Issoria lathonia	*Iss.lat*	*Zygaena filipendulae*	*Zyg.fil*
Lasiommata maera	*Las.mae*	*Zygaena lonicerae*	*Zyg.lon*
Lasiommata megera	*Las.meg*	*Zygaena viciae*	*Zyg.vic*
Lasiommata petropolitana	*Las.pet*		

Table S3. List of recorded bumblebee species and their respective abbreviations.

Species	Abbreviation	Species	Abbreviation
Bombus bohemicus	*B.boh*	*Bombus pascuorum*	*B.pas*
Bombus campestris	*B.cam*	*Bombus pratorum*	*B.pra*
Bombus distinguendus	*B.dis*	*Bombus rudedarius*	*B.rud*
Bombus hortorum	*B.hor*	*Bombus rupestris*	*B.rup*
Bombus humilis	*B.hum*	*Bombus soroeeensis*	*B.sor*
Bombus hortorum	*B.hor*	*Bombus subterraneus*	*B.sub*
Bombus jonellus	*B.jon*	*Bombus sylvarum*	*B.sylva*
Bombus lapidarius	*B.lap*	*Bombus sylvestris*	*B.sylve*
Bombus lucorum	*B.luc*	*Bombus terrestris*	*B.ter*
Bombus muscorum	*B.mus*		

Table S4. List of recorded plant species and their respective abbreviations.

Species	Abbreviation	Species	Abbreviation
Acer platanoides	*Ace.pla*	*Juniperus communis*	*Juni.com*
Achillea millefolium	*Ach.mil*	*Knautia arvensis*	*Kna.arv*
Achillea ptarmica	*Ach.pta*	*Lathyrus linifolius*	*Lat.lin*
Agrimonia eupatoria	*Agr.eup*	*Lathyrus pratensis*	*Lat.pra*
Agrostis capillaris	*Agr.cap*	*Leontodon autumnalis*	*Leo.aut*
Agrostis gigantea	*Agr.gig*	*Leucanthemum vulgare*	*Leu.vul*
Agrostis stolonifera	*Agr.sto*	*Lotus corniculatus*	*Lot.cor*
Alchemilla glabra	*Alc.glab*	*Luzula campestris*	*Luz.cam*
Alchemilla glaucescens	*Alc.glau*	*Myosotis arvensis*	*Myo.arv*
Alchemilla monticola	*Alc.mon*	*Myosotis laxa*	*Myo.lax*
Alnus glutinosa	*Aln.glu*	*Oxalis acetosella*	*Oxa.ace*
Alopecurus pratensis	*Alo.pra*	*Phleum pratense*	*Phl.pra*
Anthoxathum odoratum	*Ant.odo*	*Pimpinella major*	*Pim.maj*
Anthriscus sylvestris	*Ant.syl*	*Pimpinella saxifraga*	*Pim.sax*
Arrhenatherum pratensis	*Arr.pra*	*Plantago lanceolata*	*Pla.lan*
Betula pendula	*Bet.pen*	*Plantago major*	*Pla.maj*
Briza media	*Bri.med*	*Plantago media*	*Pla.med*
Calluna vulgaris	*Cal.vul*	*Poa annua*	*Poa.ann*
Campanula persicifolia	*Cam.per*	*Poa nemoralis*	*Poa.nem*
Campanula rotundifolia	*Cam.rot*	*Poa pratensis*	*Poa.pra*
Carex hirta	*Car.hir*	*Polygala amarella*	*Pol.ama*
Carex ovalis	*Car.ova*	*Polygala vulgaris*	*Pol.vul*
Carex pallescens	*Car.pal*	*Potentilla anserina*	*Pot.ans*
Carum carvi	*Caru.car*	*Potentilla argentea*	*Pot.arg*
Centaurea jacea	*Cen.jac*	*Potentilla erecta*	*Pot.ere*
Cerastium fontanum	*Cer.fon*	*Potentilla reptans*	*Pot.rep*
Cirsium arvense	*Cir.arv*	*Potentilla tabernaemontani*	*Pot.tab*
Convallaria majalis	*Con.maj*	*Primula veris*	*Pri.ver*
Dactylis glomerata	*Dac.glo*	*Prunella vulgaris*	*Pru.vul*
Danthonia decumbens	*Dan.dec*	*Quercus robus*	*Que.rob*
Daucus carota	*Dau.car*	*Ranunculus acris*	*Ran.acr*
Deschampsia cespitosa	*Des.ces*	*Rhinanthus minor*	*Rhi.min*
Deschampsia flexuosa	*Des.fle*	*Rosa dumalis*	*Ros.dum*
Dianthus deltoides	*Dia.del*	*Rubus idaeus*	*Rub.ida*
Elymus repens	*Ely.rep*	*Rubus saxatilis*	*Rub.sax*
Epilobium angustifolium	*Epi.ang*	*Rumex acetosa*	*Rum.acetosa*
Epilobium montanum	*Epi.mon*	*Rumex acetosella*	*Rum.acetose*
Euphrasia nemorosa	*Eup.nem*	*Rumex crispus*	*Rum.cri*
Euphrasia stricta	*Eup.str*	*Saxifraga granulata*	*Sax.gra*
Festuca ovina	*Fes.ovi*	*Sedum acre*	*Sed.acr*

Festuca pratensis	*Fes.pra*	*Sedum album*	*Sed.alb*
Festuca rubra	*Fes.rub*	*Silene nutans*	*Sil.nut*
Filipendula ulmaria	*Fil.ulm*	*Solidago virgaurea*	*Sol.vir*
Filipendula vulgaris	*Fil.vul*	*Stellaria graminea*	*Ste.gra*
Fragaria vesca	*Fra.ves*	*Stellaria media*	*Ste.med*
Fragaria viridis	*Fra.vir*	*Succisa pratensis*	*Suc.pra*
Galium boreale	*Gal.bor*	*Tanacetum vulgare*	*Tan.vul*
Galium verum	*Gal.ver*	*Taraxacum erythrosperma*	*Tan.ery*
Geranium sylvaticum	*Ger.syl*	*Taraxacum vulgare*	*Tar.vul*
Geum rivale	*Geu.riv*	*Tragopogon pratensis*	*Tra.pra*
Geum urbanum	*Geu.urb*	*Trifolium arvense*	*Tri.arv*
Glechoma hederacea	*Gle.hed*	*Trifolium hybridum*	*Tri.hyb*
Gnaphalium sylvaticum	*Gna.syl*	*Trifolium medium*	*Tri.med*
Helianthemum nummularium	*Hel.num*	*Trifolium pratense*	*Tri.pra*
Hieriacium pilosella	*Hie.pil*	*Trifolium repens*	*Tri.rep*
Hieracium Sylvaticiformia	*Hie.Syl*	*Urtica dioica*	*Urt.dio*
Hieracium Vulgatiformia	*Hie.Vul*	*Vaccinium myrtillus*	*Vac.myr*
Hypericum maculatum	*Hype.mac*	*Vaccinium vitis-idaea*	*Vac.vit*
Hypericum perforatum	*Hype.per*	*Veronica arvensis*	*Ver.arv*
Hypochaeris maculata	*Hypo.mac*	*Veronica chamaedrys*	*Ver.cha*
Juncus articulatus	*Jun.art*	*Vernonica officinalis*	*Ver.off*
Juncus compressus	*Jun.com*	*Vicia cracca*	*Vic.cra*
Juncus conglomeratus	*Jun.con*	*Vicia sepium*	*Vic.sep*
Juncus effusus	*Jun.eff*	*Viola canina*	*Vio.can*

Table S5. List of indicator plant species. The list is adapted from Eneland (2017) (See reference list in the main text).

Ajuga pyramidalis	*Gentianella campestris*	*Polygonum viviparum*
Antennaria dioica	*Gymnadenia conopsea*	*Primula farinosa*
Armeria maritima	*Helictotrichon pratense*	*Primula veris*
Arnica montana	*Hypochoeris maculata*	*Pulsatilla vulgaris*
Botrychium spp.	*Leucanthemum vulgare*	*Rhinanthus minor*
Briza media	*Linum catharticum*	*Rhinanthus serotinus*
Cardamine pratensis	*Luzula multiflora/pallidula/sudetica*	*Scorzonera humilis*
Carex hostiana	*Lychnis flos-cuculi*	*Succisa pratensis*
Carex panicea	*Nardus stricta*	*Thymus serpyllum*
Carlina vulgaris	*Ophioglossum vulgatum*	*Trifolium fragiferum*
Cirsium helenioides	*Orchis mascula*	*Trollius europaeus*
Crepis praemorsa	*Parnassia palustris*	*Veronica officinalis*
Dactylorhiza incarnata	*Pedicularis sylvatica*	*Veronica spicata*
Dactylorhiza maculata	*Pimpinella saxifraga*	*Centaurea jacea* (nectar)
Danthonia decumbens	*Pinguicula vulgaris*	*Centaurea scabiosa* (nectar)
Dianthus deltoides	*Plantago media*	*Knautia arvensis* (nectar)
Epipactis palustris	*Platanthera bifolia*	
Euphrasia spp.	*Polygala amarella*	
Filipendula vulgaris	*Polygala comosa*	
Galium verum	*Polygala vulgaris*	

Table S6. AICc values for models including explanatory variables and interactions. The lowest AICc value was used to select the model. When ΔAICc <2 the model with the interaction was selected. The best model is the first one presented for each analysis. Candidate models are organized from smallest to largest AICc. The response variable is always at the landscape scale (all habitats pooled). SNG= area of semi-natural grasslands in the landscape, PL= Presence/absence of power line corridors in the landscape, RD= road verge density in the landscape (high/low).

Response variable	Statistical model	Candidate model	AICc	ΔAICc
Number of butterfly species	Linear model (lm)	SNG + PL + RD	200.814	0
		SNG + PL + RD + PL:RD	201.713	0.899
		SNG + PL + RD + SNG:PL	203.8452	3.0312
		SNG + PL + RD + SNG:RD	203.8662	3.0522
Number of bumblebee species	Linear model (lm)	SNG + PL + RD	167.9946	0
		SNG + PL + RD + PL:RD	170.1576	2.163
		SNG + PL + RD + SNG:PL	170.886	2.8914
		SNG + PL + RD + SNG:RD	171.0435	3.0489
Number of plant species	Linear model (lm)	SNG + PL + RD	219.7634	0
		SNG + PL + RD + SNG:RD	222.4509	2.6875
		SNG + PL + RD + SNG:PL	222.6562	2.8928
		SNG + PL + RD + PL:RD	222.7001	2.9367
Number of indicator plant species	Generalized linear model (GLM) family= poisson link= log	SNG + PL + RD	141.3041	0
		SNG + PL + RD + SNG:PL	143.2122	1.9081
		SNG + PL + RD + PL:RD	143.4027	2.0986
		SNG + PL + RD + SNG:RD	144.1044	2.8003
Evenness butterflies	Linear model (lm)	SNG + PL + RD	-43.11799	0
		SNG + PL + RD + PL:RD	-40.70611	2.41188
		SNG + PL + RD + SNG:PL	-40.18513	2.93286
		SNG + PL + RD + SNG:RD	-40.08305	3.03494
Evenness bumblebees	Linear model (lm)	SNG + RD + PL	-57.38059	0
		SNG + PL + RD + SNG:PL	-56.21975	1.16084
		SNG + PL + RD + SNG:RD	-55.20015	2.18044
		SNG + PL + RD + PL:RD	-55.12685	2.25374
Evenness plants	Linear model (lm)	SNG + PL + RD + PL:RD	-172.0273	0
		SNG + PL + RD	-170.1993	1.828
		SNG + RD + PL + SNG:PL	-167.2075	4.8198
		SNG + PL + RD + SNG:RD	-167.1816	4.8457
Phylogenetic diversity butterflies	Linear model (lm)	SNG + RD + PL	78.91289	0
		SNG + PL + RD + PL:RD	80.69743	1.78454
		SNG + PL + RD + SNG:PL	81.78352	2.87063
		SNG + PL + RD + SNG:RD	81.8259	2.91301
Phylogenetic diversity bumblebees	Linear model (lm)	SNG + RD + PL	91.08421	0
		SNG + PL + RD + SNG:RD	93.3791	2.29489
		SNG + PL + RD + SNG:PL	94.13382	3.04961
		SNG + PL + RD + PL:RD	94.13606	3.05185
Phylogenetic diversity plants	Linear model (lm)	SNG + RD + PL	88.04283	0
		SNG + PL + RD + PL:RD	88.22569	0.18286
		SNG + PL + RD + SNG:PL	90.94824	2.90541
		SNG + PL + RD + SNG:RD	91.07005	3.02722

Table S7. Results of the best models given in Table S6. SNG= area of semi-natural grasslands in the landscape (continuous variable, in square kilometers), PL= Presence/absence of power line corridors in the landscape (given values are for the reference: Presence of power line corridors), RD= road verge density in the landscape (given values are for the reference: high road verge density).

Response variable	Explanatory variables	Estimate	Std.Error	t-value	Pr(>|t|)
Number of butterfly species	SNG	4.8308	9.64	0.501	0.621
	PL	-0.5073	1.7552	-0.289	0.775
	RD	-2.6307	1.7588	-1.496	0.146
Number of bumblebee species	SNG	1.5852	5.7783	0.274	0.786
	PL	0.7261	1.0510	0.691	0.495
	RD	-0.7304	1.0532	-0.693	0.494
Number of plant species	SNG	-18.912	12.975	-1.458	0.1561
	PL	6.102	2.360	2.586	0.0152*
	RD	2.074	2.365	0.877	0.3879
Number of indicator plant species (z-value is given instead of t-value)	SNG	1.2463	0.7938	1.570	0.116
	PL	0.2294	0.1565	1.466	0.143
	RD	-0.1305	0.1557	-0.838	0.402
Evenness butterflies	SNG	0.1950	0.2134	0.914	0.369
	PL	0.0008	0.0388	0.021	0.983
	RD	0.0073	0.0388	0.189	0.852
Evenness bumblebees	SNG	-0.1395	0.1708	-0.817	0.421
	RD	-0.0267	0.0311	-0.859	0.398
	PL	0.0088	0.0310	0.284	0.778
Evenness plants	SNG	0.0188	0.0302	0.620	0.5402
	PL	-0.0068	0.0072	-0.957	0.3471
	RD	-0.0138	0.0077	-1.805	0.0823
	PL:RD	0.0229	0.0108	2.109	0.0444*
Phylogenetic diversity butterflies	SNG	-1.6339	1.4365	-1.137	0.265
	RD	0.01341	0.2613	0.051	0.959
	PL	-0.3913	0.2618	-1.495	0.146
Phylogenetic diversity bumblebees	SNG	-2.1213	1.7374	-1.221	0.2323
	RD	0.5513	0.3167	1.741	0.0927
	PL	-0.1849	0.3160	-0.585	0.5630
Phylogenetic diversity plants	SNG	2.1005	1.6568	1.268	0.2153
	RD	0.1071	0.3013	0.355	0.7251
	PL	0.0853	0.3019	0.282	0.7797

Table S8. Average species richness in each landscape category for each of the studies species groups.

Landscape Category	Average species richness of butterflies (±SD)	Average species richness of bumblebees (±SD)	Average species richness of plants (±SD)	Average species richness of indicator plants (±SD)
**No Power line / High road density**	16,5 (±3)	8 (±2.8)	33 (±8.4)	4.4 (±1.7)
**No Power line / Low road density**	16.5 (±3.9)	8.6 (±2.7)	32.1 (±4.8)	5 (±1.2)
**Power line / High road density**	13.4 (±5)	8.6(±3.2)	40.3 (±6.3)	5.5 (±2.8)
**Power line/ Low road density**	18.4 (±5.5)	9.4 (±2.2)	38 (±5.5)	6 (±1.9)

Table S9. Average evenness ($E_{Shannon}$) values in each landscape category for each of the studies species groups.

Landscape Category	Average evenness of butterflies (±SD)	Average evenness of bumblebees (±SD)	Average eveness of plants (±SD)
**No Power line / High road density**	0. 79(±0.07)	0.82 (±0.08)	0.93 (±0.02)
**No Power line / Low road density**	0.74 (±0.15)	0.83 (±0.11)	0.94 (±0.01)
**Power line / High road density**	0.75 (±0.1)	0.81 (±0.09)	0.94 (±0.01)
**Power line/ Low road density**	0.77 (±0.06)	0.86 (±0.04)	0.93 (±0.01)

Table S10. Average standardized effect size of phylogenetic diversity values in each landscape category for each of the studies species groups.

Landscape Category	Average standardized effect size of phylogenetic diversity of butterflies (±SD)	Average standardized effect size of phylogenetic diversity of bumblebees (±SD)	Average standardized effect size of phylogenetic diversity of plants (±SD)
**No Power line / High road density**	-1.2 (±0.51)	0.1 (±1.02)	-0.4 (±0.9)
**No Power line / Low road density**	-0.4 (±0.94)	-0.2 (±0.68)	-1.1 (±0.84)
**Power line / High road density**	-0.8 (±0.57)	0.1 (±0.88)	-0.9 (±0.47)
**Power line/ Low road density**	-0.7 (±0.6)	-0.5 (±0.76)	-0.5 (±0.76)
